# Interprofessional Leadership Development: Role of Emotional Intelligence and Communication Skills Training

**DOI:** 10.15766/mep_2374-8265.11247

**Published:** 2022-05-13

**Authors:** Sneha Shrivastava, Johanna Martinez, Daniel J. Coletti, Alice Fornari

**Affiliations:** 1 Assistant Professor of Medicine, Department of Medicine, Donald and Barbara Zucker School of Medicine at Hofstra/Northwell; 2 Associate Professor of Medicine, Department of Medicine, Donald and Barbara Zucker School of Medicine at Hofstra/Northwell; 3 Associate Professor, Department of Psychiatry and Medicine, Donald and Barbara Zucker School of Medicine at Hofstra/Northwell; 4 Professor of Medicine, Donald and Barbara Zucker School of Medicine at Hofstra/Northwell

**Keywords:** Emotional Intelligence, Communication Skills, Interprofessional Education, Leadership Development, Patient Safety

## Abstract

**Introduction:**

Among the many skills required for leading interprofessional health care teams, emotional intelligence and communication skills are critical to building professionalism, establishing patient trust, and providing optimal patient care. Nonetheless, these skills are often overlooked in medical training. We implemented a 2.5-hour workshop for interprofessional trainees to self-assess, reflect, and apply their emotional intelligence and communication skills.

**Methods:**

Participants were interprofessional trainees, including internal medicine residents, medical students, and graduate students in clinical pharmacy, physician assistant, and health psychology training programs. The workshop consisted of reflective activities to self-assess emotional intelligence and communication styles; a didactic presentation focused on leadership, emotional intelligence, and communication styles; and a teamwork activity to apply emotional intelligence and communication skills.

**Results:**

Forty-four trainees participated in this workshop. After the workshop, trainees reported increased knowledge about positive strategies to communicate with team members, felt more comfortable working with other professionals to encourage positive team dynamics, and were more prepared to encourage leadership in their interprofessional teams. Examination of learner evaluations suggested that residents endorsed higher mean ratings than the other learner groups in knowledge attainment (*p* = .02) and meeting all learners' needs (*p* = .01).

**Discussion:**

This workshop enhanced our trainees' self-reported comfort, awareness, and preparedness regarding using emotional intelligence and communication strategies. An interprofessional approach can be beneficial for leadership training in the health professions.

## Educational Objectives

By the end of this activity, learners will be able to:

1. Describe the behavioral components of leadership.
2. Self-assess their emotional intelligence and communication styles.
3. Apply emotional intelligence and communication skills in a team-based scenario.
4. Reflect on emotional intelligence and communication skills needed for working in interprofessional teams.

## Introduction

To promote interprofessional practice and education (IPE) and prepare health professionals to succeed in interprofessional teams, the Interprofessional Education Collaborative (IPEC), representing 21 national health professional associations, developed core competencies required for interprofessional collaborative practice.^1,2^ The Accreditation Council for Graduate Medical Education (ACGME) and the Liaison Committee on Medical Education (LCME) recommend integrating interprofessional education into medical curricula to prepare learners for interprofessional collaboration in their future practices.^3,4^ Incorporating interprofessional experiences early in health care professionals' training prepares learners to work efficiently in teams and deliver effective patient care during and after their education. A 2013 Cochrane Review documented the positive effects of interprofessional education on diabetes care, interventions to address domestic violence, patient satisfaction, collaborative team behavior, and patient care.^5^ Interprofessional education also positively impacts student perceptions of and attitudes toward working collaboratively.^6^

Despite these benefits and the recommendations from the IPEC, ACGME, and LCME, programs at the undergraduate and graduate levels often lack a robust interprofessional curriculum.^7^ Limited financial resources and support at institutions, scheduling IPE delivery, incompatible health professional degree calendars, and differences in assessment requirements have been attributed as significant barriers influencing IPE at an institutional level.^8^ Heavy faculty teaching and administrative workloads, limited knowledge about other health professional education, lack of perceived value of IPE, and student learning styles also influence the development of IPE curricula.^8^

We have an interprofessional clinical team model that provides primary care for our institution's complex internal medicine patients. Trainees and faculty from different health professions backgrounds assigned to this model do not have prior training on working in or leading interprofessional teams. Our primary care practice model requires high-performing interprofessional teams to care for medically complex patients; therefore, we designed a series of IPE workshops to meet this need and introduce interprofessional care skills. We created this workshop to support the core competencies of emotional intelligence (EI) and communication skills, as we believe these abilities are essential to functioning in interprofessional teams.

Effective interprofessional teams also require skilled leaders. Many trainees assume roles as frontline team leaders at some point in their careers. Leadership competencies are multidetermined. Effective leaders have a high degree of EI^9^ and excel in communication skills.^10^ EI contributes to 80% of a leader's success.^11^ Contrary to prior beliefs, EI and communication skills can be measured, taught, learned, enhanced, and incorporated into medical education.^12^ EI can help trainees master the ACGME competencies of professionalism and interpersonal and communication skills.^13^

The benefits of EI and communication training have been demonstrated across all health professions. There is a positive correlation between medical education and EI competency.^14^ Development of EI skills has been associated with improved trainee academic and clinical performance.^15,16^ Communication skills work synergistically with EI skills. Along with EI abilities, communication skills can also improve patient safety among trainees.^17^

While several prior studies have focused on leadership training in the health professions, only a few have taken an interprofessional approach.^18,19^ Previous *MedEdPORTAL* publications on leadership curricula have been tailored to medical students, residents, and faculty training.^20–24^ Interprofessional curricula with leadership and communications skills components have been limited to training emergency medical teams in hospitals.^25,26^ Since interprofessional teams operate in all aspects of health care, we created an interprofessional leadership workshop generalizable to all health care settings (ambulatory and hospital) and for all interprofessional health care trainees. Understanding that EI and communication skills are essential for successful interprofessional clinical care and leadership, we designed a workshop focused on these core skills. The goals of the workshop were to (1) describe the behavioral components of leadership, (2) self-assess EI and communication style, (3) apply EI leadership in a team-based clinical scenario, and (4) reflect on the EI and communication skills needed for working in interprofessional teams. Here, we describe our experience designing, delivering, and evaluating this IPE workshop and present its results and impact on our trainees.

Interprofessional education lacks a gold-standard theoretical framework.^27^ The conceptual framework we used is based on the IPEC core competencies for collaborative practice.^2^ The competencies intend to build on each profession's expected competencies for interprofessional collaborative practice; therefore, they are a natural model to support our educational and practice efforts. The IPEC panel identifies four core competency domains: (1) values and ethics, (2) roles and responsibilities for collaborative practice, (3) interprofessional communication, and (4) teamwork and team-based care. As designed and implemented, our workshop addresses all four competency domains.

Our theoretical framework is based on an educational model in which learning together as an interprofessional team aligns with the skills needed to practice together in the clinic. Social and situated learning theory supports our educational model. This workshop has been designed to include observation and modeling opportunities, which are key characteristics of social learning theory, as well as activities that support an interplay among learners and faculty.^28,29^ Our intent to focus on the IPEC competencies to frame content and the core principles of situated and social learning theory has resulted in a successful learning model, practice with interprofessional peers, and building a community of practice through social interaction.^30^

## Methods

### Target Audience/Setting

Our target audience was learners working together in an interprofessional clinical care team at a single ambulatory clinical site. They included internal medicine residents, medical students, and graduate students in clinical pharmacy, physician assistant (PA), and health psychology trainees. The workshop was conducted in a conference room with group seating for seven to 10 learners per table to facilitate discussion during paired learner and small-group activities. This workshop was among a series of educational didactics created for our interprofessional trainees, and their attendance was mandatory.

### Instructors

Our workshop was facilitated by two lead faculty, a physician and a medical education expert, who had prior EI and communication skills training to support interprofessional leadership. Faculty from each profession (internal medicine, clinical pharmacy, PA, and health psychology) served as small-group facilitators for the workshop.

### Preparation

Facilitators reviewed all resource and appendix files (Appendices A–D) before the workshop. Appendix A (the PowerPoint presentation) provided references, notes, and suggested workshop talking points. Facilitators selected a location that allowed for small- and large-group discussions. An online platform with breakout rooms could work if designated faculty or staff are comfortable with the technology platform. Before the workshop, the facilitators printed the EI self-assessment tool^31^ and the communication style inventory^32^ for distribution to each participant. If this workshop was being conducted in an online setting, these materials and assessment tools could be provided to participants electronically as online surveys.

### Implementation

The facilitators introduced the workshop (Appendix A, slides 1–4) and the IPEC competencies, which aligned with the workshop's interprofessional education themes. This introduction was framed as a reminder to participants of the importance of interprofessional education and its direct impact on interprofessional clinical teams. All the participants completed the EI^31^ and communication style inventory^32^ self-assessment tools and calculated their scores using the rubrics outlined in the questionnaires (15 minutes). The EI self-assessment tool^31^ used in our workshop, developed by the University of Minnesota Extension, is no longer available. We recommend using the freely available Self-Report Emotional Intelligence Test,^33^ which has been widely used and validated within health professions education. The communication style inventory that we used was obtained from the book *I-Speak Your Language: A Survey of Personal Styles,* by Michael Bednarski, which is frequently used in corporate training programs.^32^ Facilitators could also consider using other, more freely accessible instruments, such as the Communication Styles Quiz and research by Leadership IQ,^34^ to enable participants to identify their communication styles. Although these communication tools have not been validated for use in medicine, they could allow participants to gain insight into their communication style.

The workshop facilitators conducted an icebreaker exercise in a large-group format to encourage participants to brainstorm and identify the skills required to make a great leader (Appendix A, slide 6). A framing talk was given on the importance of EI and communication in leadership, and the key components of EI were described (Appendix A, slides 7–16). Participants worked in pairs to review and discuss their EI scores, identify their EI strengths and weaknesses, and determine the specific EI components they wished to improve. The facilitators then discussed the different communication styles and outlined the strengths and challenges inherent in each style (Appendix A, slides 18–24).^32^ Participants worked in pairs to discuss their predominant communication styles, reflect on the challenges of communicating with a difficult team member, and improve their communication within their teams. The didactic and small-group work took 60 minutes in total.

A fishbowl activity (40 minutes) was conducted in small groups to apply communication skills and EI constructs in understanding team dynamics (Appendix B). Each small group was separated into an inner and an outer circle. The inner circle participants engaged in problem-solving, and the outer circle observed the inner circle's interactions.^35^ One of the outer circle participants was assigned to be the observer. Each small group had a faculty facilitator who was a faculty member at our interprofessional clinic.

Inspired by a challenge our training program was facing at the time, the inner circle was assigned the task of designing an ideal meeting space for an interprofessional team—conducive to both clinical huddling and educational sessions. Participants were asked to consider the architectural layout of the room, ambiance, learning climate, seating, social connectivity, patient confidentiality, and IT resources. The observer was tasked with identifying the inner circle participants' communication and EI skills using the observer checklist (Appendix C). We developed the checklist to mirror the EI skills discussed earlier in the workshop and included individual behaviors and team dynamics items. Although it has not been validated, the checklist can guide the assigned observer to recognize the EI and communication styles and share them in the debrief.

Upon completing the fishbowl activity, the outer circle participants reported their findings on the group's communication and EI skills. The faculty facilitators debriefed their small groups and provided feedback on the EI and communication skills displayed (15 minutes).

To conclude the workshop, participants were shown a publicly available video depicting a new version of the old tortoise and hare story that emphasized the importance of communication and EI skills needed for teamwork and leadership.^36^ Participants completed a reflective workshop evaluation (Appendix D; 20 minutes).

### Workshop Evaluation

The workshop evaluation included quantitative and qualitative items to gauge participants' knowledge and attitudes after the workshop.^37^ Specifically, participants were asked to describe (1) a take-home message, (2) a skill they had learned, and (3) a concept that remained unclear. Participants also provided general feedback or comments on their experiences during the session. They used an 11-point Likert scale (0 = *don't agree at all,* 10 = *completely agree*) to rate their perceived comfort in applying EI and communication styles to improve their leadership qualities and interaction within their interprofessional teams in the clinical setting. We purposefully chose a broader 11-point Likert-scale range similar to Thurstone's seminal work on attitude assessment.^38^ We made this decision to optimize precision and potential range of responses, guided by research suggesting there was no negative time or bias associated with larger scales when the goal was to invite a wide range of responses.^39^

Content analysis was performed on the open-ended responses by two authors, and themes were derived from the data using a consensus process to reach the final themes. Nonparametric analyses comparing distributions of responses (i.e., Mann-Whitney *U* tests) were deployed to examine differences among the learners for Likert-scale responses. These analyses were completed using the Statistical Package for the Social Sciences, version 22 (IBM).

The workshop was determined by our institutional review board to constitute a quality improvement initiative.

## Results

Sixty-one participants attended the workshop. Among the attendees were 15 interprofessional faculty members and 44 learners, including medical students (*n* = 22 or 50%; nine in year 1, nine in year 2, two in year 3, and two in year 4), internal medicine residents (*n* = 9 or 21%), sixth-year pharmacy interns (*n* = 10 or 23%), one PA student, and one psychology extern. Participant postworkshop reflections and feedback are outlined in the Table. The most common themes derived from the responses involved observations that “leadership is a skill” and that “communication is an important aspect of teamwork.” Participants' most common response to describing a new skill they had learned was “the different communication styles.” The most common response to describing something they still found confusing was “how the different communication styles could affect leadership.”

**Table.**
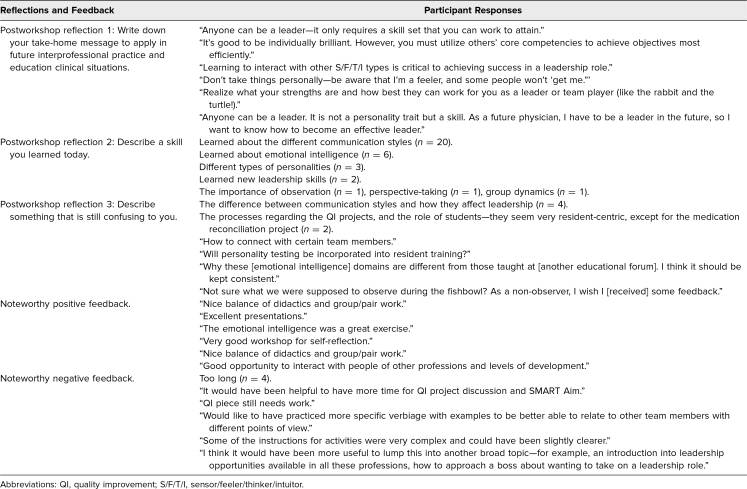
Participants' Postworkshop Reflections and Feedback

Most participants found the workshop topic and the activities enjoyable, engaging, and valuable. Examination of the Likert-scale responses suggested that residents endorsed higher mean ratings than the other learner groups in self-awareness for communication (*p* = .02) and felt that the workshop met the needs of all learners (*p* = .01; Figure). At the end of the workshop, all trainees reported increased comfort, awareness, and preparedness with regard to using EI and communication style to improve leadership.

**Figure.**
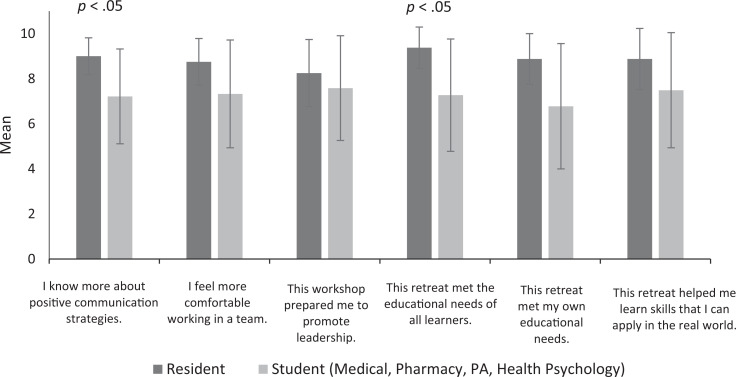
Learner evaluation of the workshop. Error bars indicate standard deviation. Abbreviation: PA, physician assistant.

## Discussion

This workshop exposed our trainees to the construct of EI and the importance of communication skills in working effectively in interprofessional teams. The workshop was the first opportunity for most of our trainees to evaluate their EI and communication skills. Encouraging self-reflection on their EI and communication skills scores allowed learners to understand the dynamics of working in interprofessional teams in busy ambulatory clinical environments, an opportunity not typically feasible at their training sites. Furthermore, the fishbowl activity enabled learners to practice their EI and communication skills and receive peer and faculty feedback, a strategy supported by social learning theory. Trainees felt that this workshop increased their self-awareness of EI and communication skills and thought they could apply them in their clinical settings.

The difference in the training levels of participants, such as residents versus more junior learners, may have influenced their depth of understanding of the leadership skills and ability to apply these constructs to real-world challenges within interprofessional teams. For example, most of the trainees were medical students in their first 2 years of medical school. They may not have fully understood either their leadership potential at that early stage of their medical training or the impact of communication skills on leadership. In contrast, it is possible that licensed learners (our internal medicine residents) were more able to benefit from the workshop due to their greater level of clinical experience and familiarity with interprofessional team functioning. Examination of select qualitative comments from our medical and pharmacy students indicated that some unlicensed learners felt they lacked sufficient real-world clinical experience to apply their new EI skills in situations presented during our group activities.

The positive evaluation responses to our workshop were similar to those of other health professions educational training focusing either on EI,^40,41^ communication,^42,43^ or both EI and communication.^42,44,45^

A major limitation of this workshop is that the participants practice clinical care at only one academic ambulatory care site. While several different communication and EI self-assessment tools are available, resource limitations precluded the use of proprietary EI and communication self-assessment tools. The EI^31^ and communication style^32^ self-assessment tools that we used are not validated for medical professional use.

It would have been helpful to include health professions trainees across all levels of training, and their feedback could have been useful in replicating this work outside our institution. The workshop evaluation may not accurately represent PA and health psychology students' views since only one PA student and one health psychology student participated in the workshop. One of the challenges of constructing an interprofessional training team involves matching learner training needs and patient care needs. We believe that one psychology extern can meet the behavioral health needs of our primary care patients, as only one extern practices at our ambulatory care clinic at any time. We want to note that the extern is always highly valued by other team members, is frequently sought out to participate in patient care, and perceives themselves to be included and welcome. That notwithstanding, an equivalent number of trainees from each profession would optimize the small-group activities.

This interprofessional workshop was created, edited, and reviewed in an interprofessional manner by a team including health professional educators, internal medicine faculty, and an interprofessional education committee within our general internal medicine division. Our institution's interprofessional team consisted of trainees from different levels of training, ranging from internal medicine residents to medical students to graduate students from various programs. Since these trainees worked together often, it was essential to include all of them in the workshop, which benefited from the prior interactions some learners had had with each other in our clinical training setting. Although our institution's interprofessional team composition might differ from other institutions', this learner-centric workshop can be adapted to all health professions, at all levels of prior clinical experience, and involve learners who have little prior interaction as well as those who are already members of clinical teams.

Lessons learned from the implementation of the workshop include the importance of protected time for extended interactive education with peers and faculty, despite faculty schedule conflicts and conference space limitations. Addressing the challenge of delivering collaborative interprofessional education that brings together interprofessional trainees and professionals must be a priority of leadership and faculty, as it connects education to practice, a goal for all educators. Despite scheduling challenges, this workshop demonstrates that the ideal conceptual framework of interprofessional learning can be accomplished. The workshop can be adapted to any online communication and learning platform for institutions that may have limited space or to optimize time, minimize travel, or practice social distancing. The virtual transformation would benefit from additional technical support staff, consistent with most education transformations to a virtual environment. A retrospective pre/post evaluation would have been helpful in assessing participants' comfort with, awareness of, and preparedness for the skills taught in the workshop.

Future directions include advancing the workshop to account for trainees' progression in EI and communication skills over time. It is important for faculty leadership to consider how to help learners develop these skills longitudinally for professional growth, which would be evidenced by the act of doing and role-modeling the skills while interacting with others.

There has been a shift in the culture of medicine to incorporate an interprofessional approach to patient care. Since our learners need to work in interprofessional teams, accrediting bodies should consider including leadership as a core competency for all health professional learners. This workshop can be used to introduce, discuss, and practice leadership skills for IPE.

## Appendices


EI in Interprofessional Leadership.pptxFacilitator Guide to Fishbowl Activity.docxSmall-Group Fishbowl Activity Evaluation.docxWorkshop Evaluation.docx
*All appendices are peer reviewed as integral parts of the Original Publication.*
