# Supplementary material for: Interprofessional Leadership Development: Role of Emotional Intelligence and Communication Skills Training
Source: MedEdPORTAL. 2022 May 13;18:11247. doi: 10.15766/mep_2374-8265.11247 (PMC9098732; doi:10.15766/mep_2374-8265.11247)
Supplement: Supplementary file 1 — EI in Interprofessional Leadership.pptxFacilitator Guide to Fishbowl Activity.docxSmall-Group Fishbowl Activity Evaluation.docxWorkshop Evaluation.docx [file mep_2374-8265.11247-s001.zip › B. Facilitator Guide to Fishbowl Activity.docx]

**Appendix B: Facilitator Guide to Fishbowl Activity**

SEGMENT OVERVIEW:

- Participants will complete a 30-minute discussion in a fishbowl format^1^, followed by a 15-minute debrief. The fishbowl activity can occur face to face or virtually if necessary.
- All participants will be divided into small groups of 8-10 participants, allowing for multiple fishbowl discussions to run simultaneously. Each small group will be divided into inner and outer circle participants, and their chairs can be re-arranged to form the two circles, inner and outer, in each small group. In an online format, this can be accomplished with breakout rooms and strategically turning on cameras to focus on either the inner or outer circle.
- Each small group will have inner circle participants, outer circle participants, an observer, and a faculty facilitator.
  - The role of inner circle participants is to discuss the design of an ideal meeting set-up for an interprofessional team conducive to clinical huddling and educational sessions.
  - The role of outer circle participants is to actively listen and identify emotional intelligence skills and communication styles in the inner circle discussion.
  - The role of the observer is to actively observe the inner circle discussion and complete an evaluation form to assess individual behaviors, team dynamics and identify the inner circle participants' communication and emotional intelligence skills (Appendix C).
  - The role of the faculty is to facilitate the discussion and lead the feedback and debrief segment.
- Suggestions to perform the fishbowl activity for an online format.
  - Multiple breakout rooms can be created to run multiple small group fishbowl discussions simultaneously.
  - To focus on inner circle discussion, inner circle participants can turn on their camera, and outer circle participants can turn off their camera.
  - To focus on feedback from the outer circle, outer circle participants can turn on their camera, and inner circle participants can turn off their camera.
- Suggestions for a topic of discussion
  - We suggest choosing a general topic for an organic discussion that does not focus on the specialized knowledge of any particular health profession and does not need faculty facilitation.
  - The topic we chose for discussion was the design of an ideal meeting set-up for an interprofessional team.
  - We recommend providing the participants' discussion points or subtopics to structure the discussion.
  - We asked participants to consider the architectural layout of the room, ambiance, learning climate, seating, social connectivity, patient confidentiality, and IT resources. While discussing the elements of an interprofessional room set-up, participants had opportunities to display the different components of emotional intelligence (self-awareness, motivation, empathy, social skills, and self-regulation) and the different communication styles (intuitor, thinker, feeler sensor).

DETAILS ON FLOW:

- **Setting the stage (5 minutes):**
  - In a large group format, the lead workshop facilitator(s) can introduce the fishbowl activity, goals, and the main problem for discussion.
  - Divide the workshop participants into small groups, with 8-10 participants per group. Assign one faculty for each small group to facilitate the discussion and debrief.
  - Once in small groups, faculty facilitators ask participants to be in an inner circle or outer circle.
  - Among the outer circle participants, ask for a volunteer to be an observer. Share the checklist with the observer and give them time (5 minutes) to read and ask any questions if they have.
- **30-minute inner circle discussion**
  - Once all roles are assigned, inner group participants start discussing the chosen topic for 30 minutes.
  - Small group faculty facilitators can check in with the observer (in-person or via chat function is using an online platform) to answer any questions.
  - Small group facilitators can keep track of time and announce when 5 minutes remain in the discussion.
  - After 30 minutes of discussion, announce the end of discussion and immediately begin the debrief session
- **15-minute debrief**
  - The small group faculty facilitator will start the debrief by explaining the debrief protocol. The faculty will start by eliciting from the outer circle participants their perceptions of how the interaction went.
  - The observer can then share their specific feedback based on the observation checklist.
  - During this discussion, faculty should encourage the participants to share the specific observed emotional intelligence skills and communication styles.
  - Encourage the participants to share any aspects of the discussion felt were challenging.
  - Small group faculty facilitators should reserve the last 2 minutes of the debrief to ask the participants to share their take-home points from the activity.

OBSERVER TRAINING:

- The observer checklist is a one-page checklist that mirrors the EI skills discussed earlier in the workshop and include items on individual behaviors and team dynamic.
- Observers can be given time (5 minutes) to read and understand the checklist.
- The small group faculty facilitator can help observers by answering their questions before and during the discussion.

FACULTY TRAINING:

- One faculty facilitator per small group faculty should be recruited before the workshop. We suggest identifying interested faculty at least one month ahead of time to secure their time.
- Lead workshop facilitators can share Appendix A, B, and C with faculty before the workshop to help in their preparation.
- If needed, a faculty development session can be conducted before the workshop to highlight the different emotional intelligence skills and communication styles, explain the goals and outline of the workshop, the fishbowl activity, their role in helping the observer, and lead the debrief.
- Below are some suggestions that might help faculty with debriefing.

**QUESTIONS FOR CONSIDERATION TO GUIDE FACULTY DEBRIEF**

**Faculty should let participants give feedback, reflect guide the debrief. There are 3 domains around which faculty can provide feedback.**

1) Provide feedback on the **emotional intelligence skills** displayed in the inner circle discussion (see Appendix C).

- You can provide feedback from the perspective of the participant.
- Did the participants recognize opportunities for using any emotional intelligence skills?
- Did the participants use the core emotional intelligence skills of self-awareness, motivation, empathy, social skills, and self-regulation?

2) Provide feedback on the **communication styles** displayed in the inner circle discussion (see Appendix C).

- What were the communication styles the participants were using?
- Were the participants able to identify if they were a thinker, feeler, sensor, or intuitor?
- Were participants able to change and adapt their style based on the discussion?

3) Provide an opportunity for the participants to share their observations (see Appendix C). Examples of some questions include:

- Can you share the emotional intelligence skills with examples you observed during the discussion?
- How would aspects of the discussion improve if participants chose a different communication style?

Reference

1. Jaques D. Teaching small groups. *BMJ*. 2003;326(7387):492-494. DOI: 10.1136/bmj.326.7387.492.
