# Supplementary material for: Interprofessional Leadership Development: Role of Emotional Intelligence and Communication Skills Training
Source: MedEdPORTAL. 2022 May 13;18:11247. doi: 10.15766/mep_2374-8265.11247 (PMC9098732; doi:10.15766/mep_2374-8265.11247)
Supplement: Supplementary file 1 — EI in Interprofessional Leadership.pptxFacilitator Guide to Fishbowl Activity.docxSmall-Group Fishbowl Activity Evaluation.docxWorkshop Evaluation.docx [file mep_2374-8265.11247-s001.zip › D. Workshop Evaluation.docx]

**Appendix D: Workshop Evaluation**

I am a (check one):

| 🞏 Medical Resident | 🞏 Medical Student |
| --- | --- |
| 🞏 Pharmacy Resident | 🞏 PA Student |
| 🞏 Pharmacy Student | 🞏 Faculty/Attending 🞏 Support Team |
| 🞏 Psychology Extern | 🞏 Other (_____________) |

Write down your “take-home message” from this retreat that you can apply to clinical situations.

Describe a skill that you learned today and how it might be useful to you in your work:

Describe something covered today that is ***confusing*** to you or unclear:

General Comments or Feedback [any constructive feedback, welcome!]:

AFTER PARTICIPATING IN TODAY’S RETREAT…

***I know more* about how to use positive strategies to communicate with patients/team members:**

0 1 2 3 4 5 6 7 8 9 10

Don’t agree Agree a Agree a moderate Completely

at all a little amount Agree

***I feel more comfortable* working with other professionals to encourage positive team dynamics.**

0 1 2 3 4 5 6 7 8 9 10

Don’t agree Agree a Agree a moderate Completely

at all a little amount Agree

**I think this workshop *made me more prepared* to promote leadership in my program**

0 1 2 3 4 5 6 7 8 9 10

Don’t agree Agree a Agree a moderate Completely

at all a little amount Agree

**In my opinion…**

**This retreat met the educational needs *of all the learners from each profession*.**

0 1 2 3 4 5 6 7 8 9 10

Don’t agree Agree a Agree a moderate Completely

at all a little amount Agree

**This retreat met *my own* educational needs.**

0 1 2 3 4 5 6 7 8 9 10

Don’t agree Agree a Agree a moderate Completely

at all a little amount Agree

**This retreat helped me learn skills that I can apply in the “real world.”**

0 1 2 3 4 5 6 7 8 9 10

Don’t agree Agree a Agree a moderate Completely

at all a little amount Agree
