# Supplementary material for: Interprofessional Leadership Development: Role of Emotional Intelligence and Communication Skills Training
Source: MedEdPORTAL. 2022 May 13;18:11247. doi: 10.15766/mep_2374-8265.11247 (PMC9098732; doi:10.15766/mep_2374-8265.11247)
Supplement: Supplementary file 1 — EI in Interprofessional Leadership.pptxFacilitator Guide to Fishbowl Activity.docxSmall-Group Fishbowl Activity Evaluation.docxWorkshop Evaluation.docx [file mep_2374-8265.11247-s001.zip › A. EI in Interprofessional Leadership.pptx]

## Slide 1
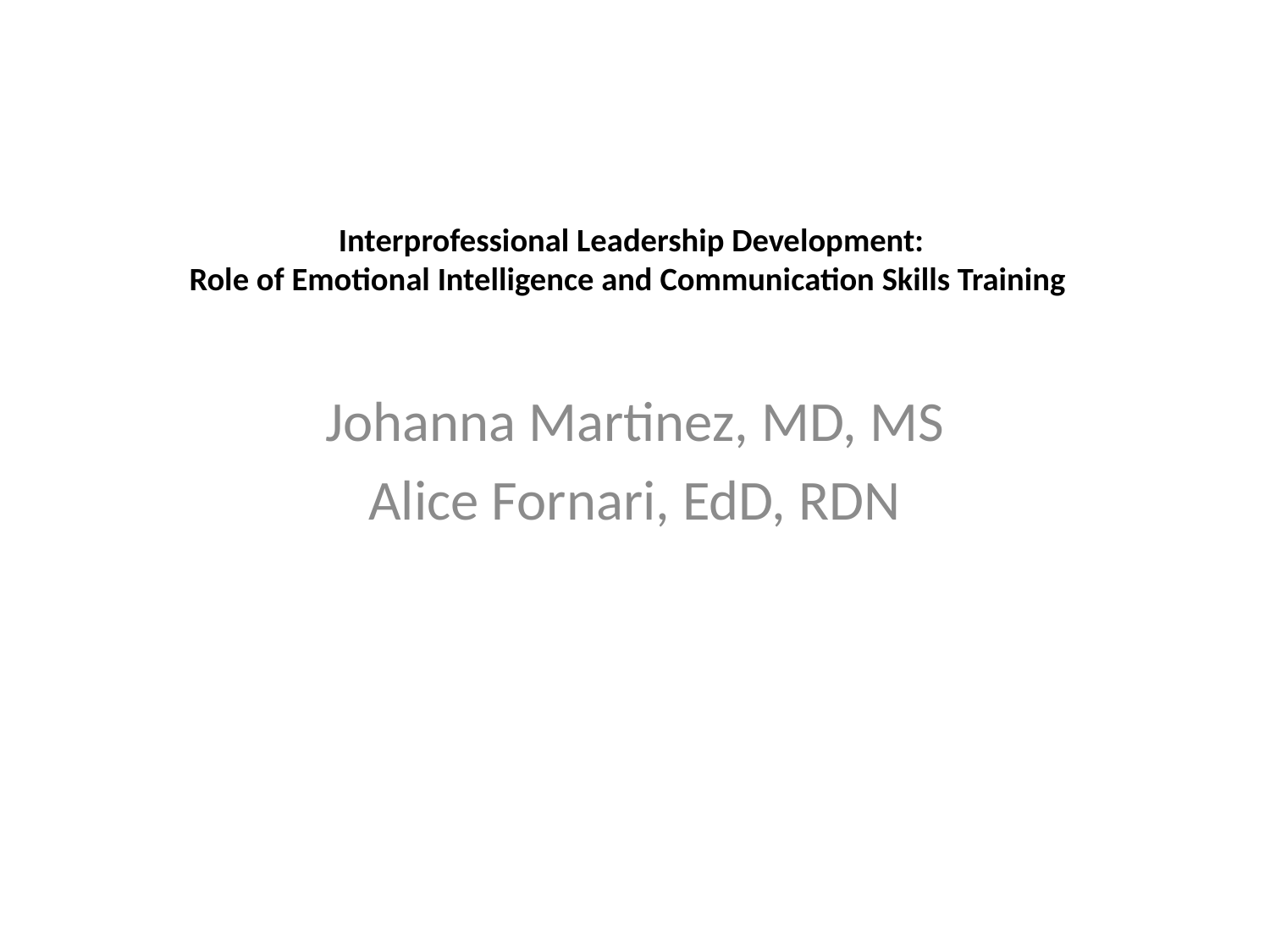

# Interprofessional Leadership Development: Role of Emotional Intelligence and Communication Skills Training
Johanna Martinez, MD, MS
Alice Fornari, EdD, RDN

## Slide 2
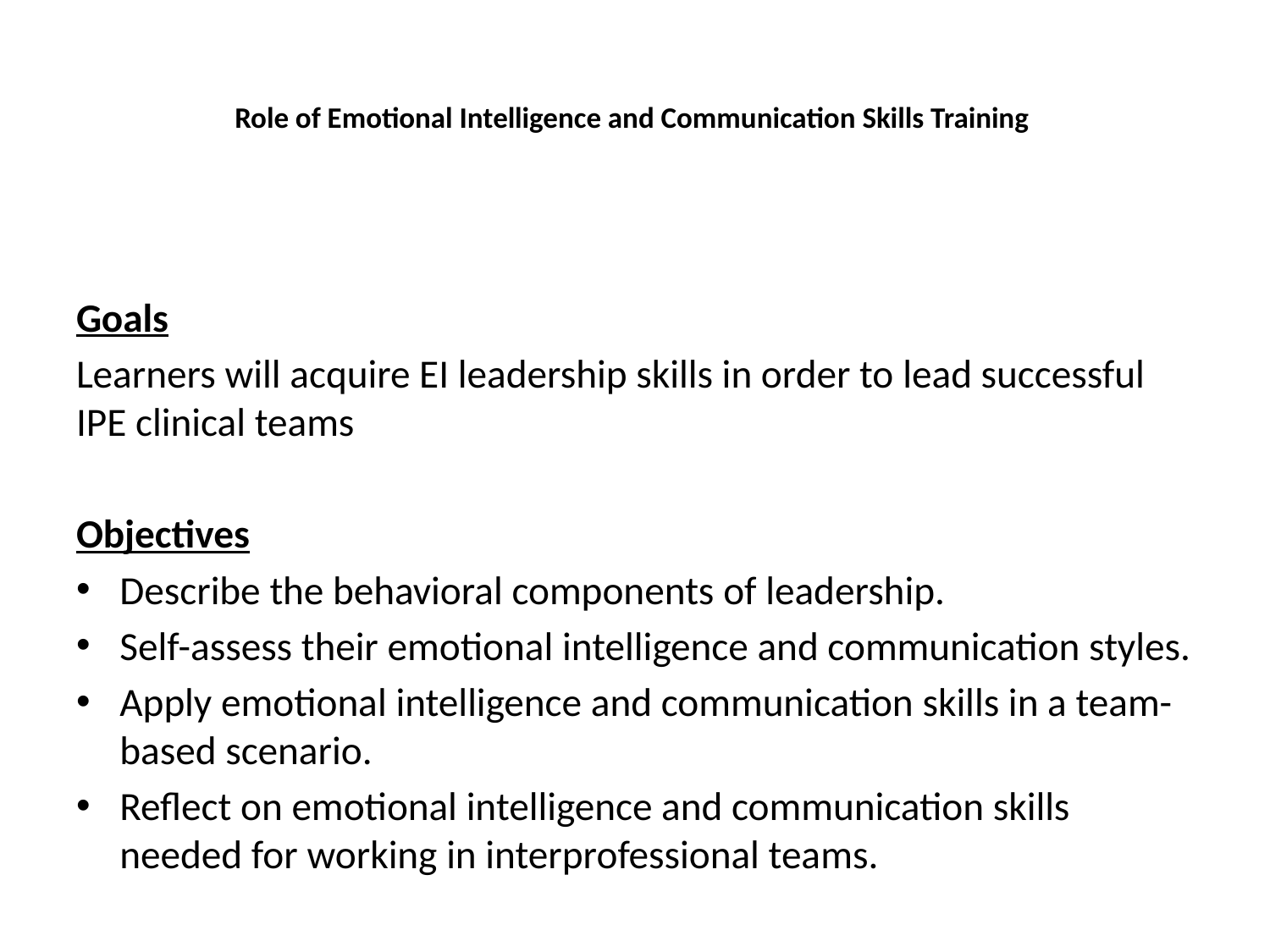

# Role of Emotional Intelligence and Communication Skills Training
Goals
Learners will acquire EI leadership skills in order to lead successful IPE clinical teams
Objectives
Describe the behavioral components of leadership.
Self-assess their emotional intelligence and communication styles.
Apply emotional intelligence and communication skills in a team-based scenario.
Reflect on emotional intelligence and communication skills needed for working in interprofessional teams.

## Slide 3
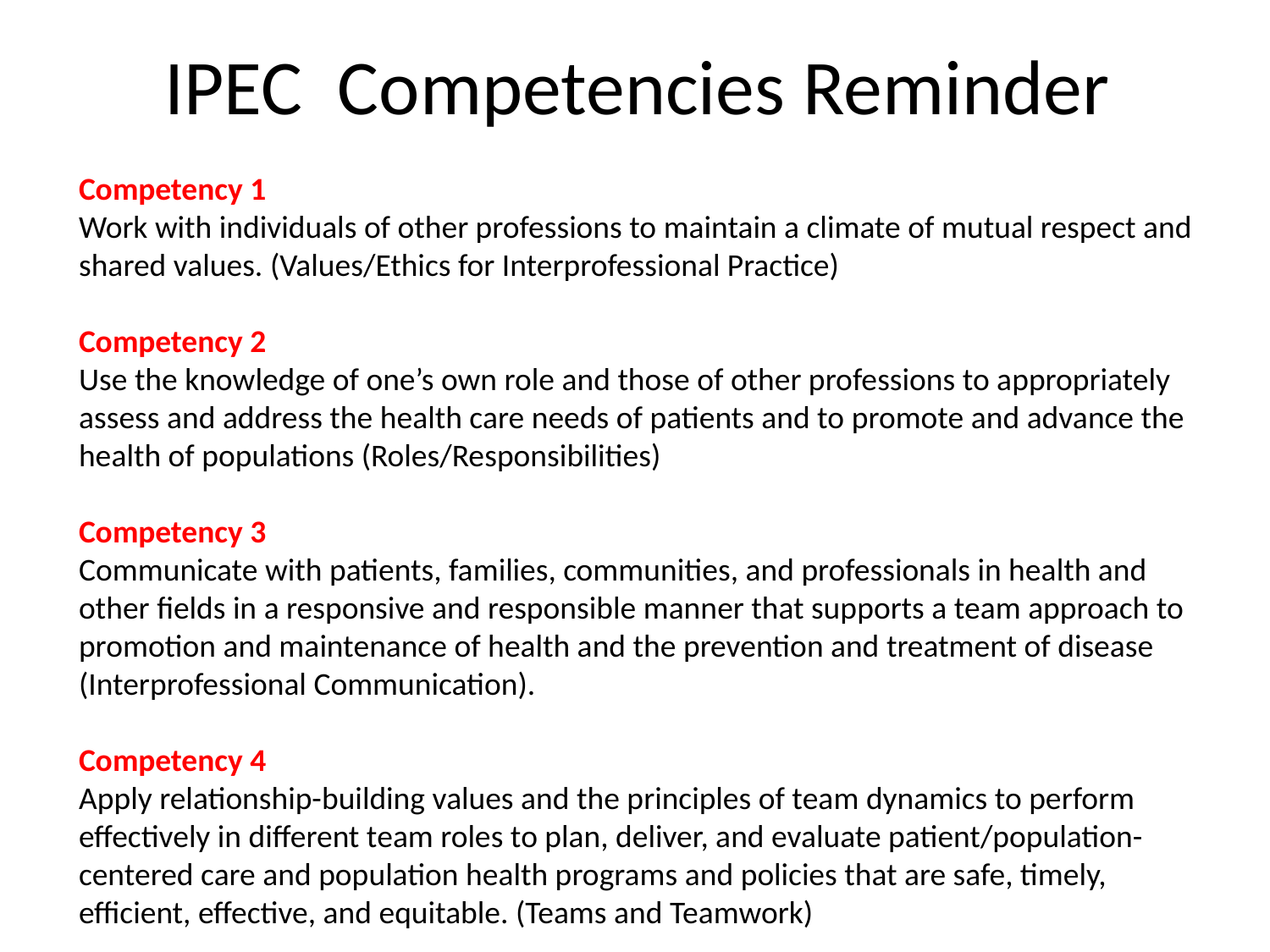

# IPEC Competencies Reminder
Competency 1
Work with individuals of other professions to maintain a climate of mutual respect and shared values. (Values/Ethics for Interprofessional Practice)
Competency 2
Use the knowledge of one’s own role and those of other professions to appropriately assess and address the health care needs of patients and to promote and advance the health of populations (Roles/Responsibilities)
Competency 3
Communicate with patients, families, communities, and professionals in health and other fields in a responsive and responsible manner that supports a team approach to promotion and maintenance of health and the prevention and treatment of disease (Interprofessional Communication).
Competency 4
Apply relationship-building values and the principles of team dynamics to perform effectively in different team roles to plan, deliver, and evaluate patient/population-centered care and population health programs and policies that are safe, timely, efficient, effective, and equitable. (Teams and Teamwork)

## Slide 4
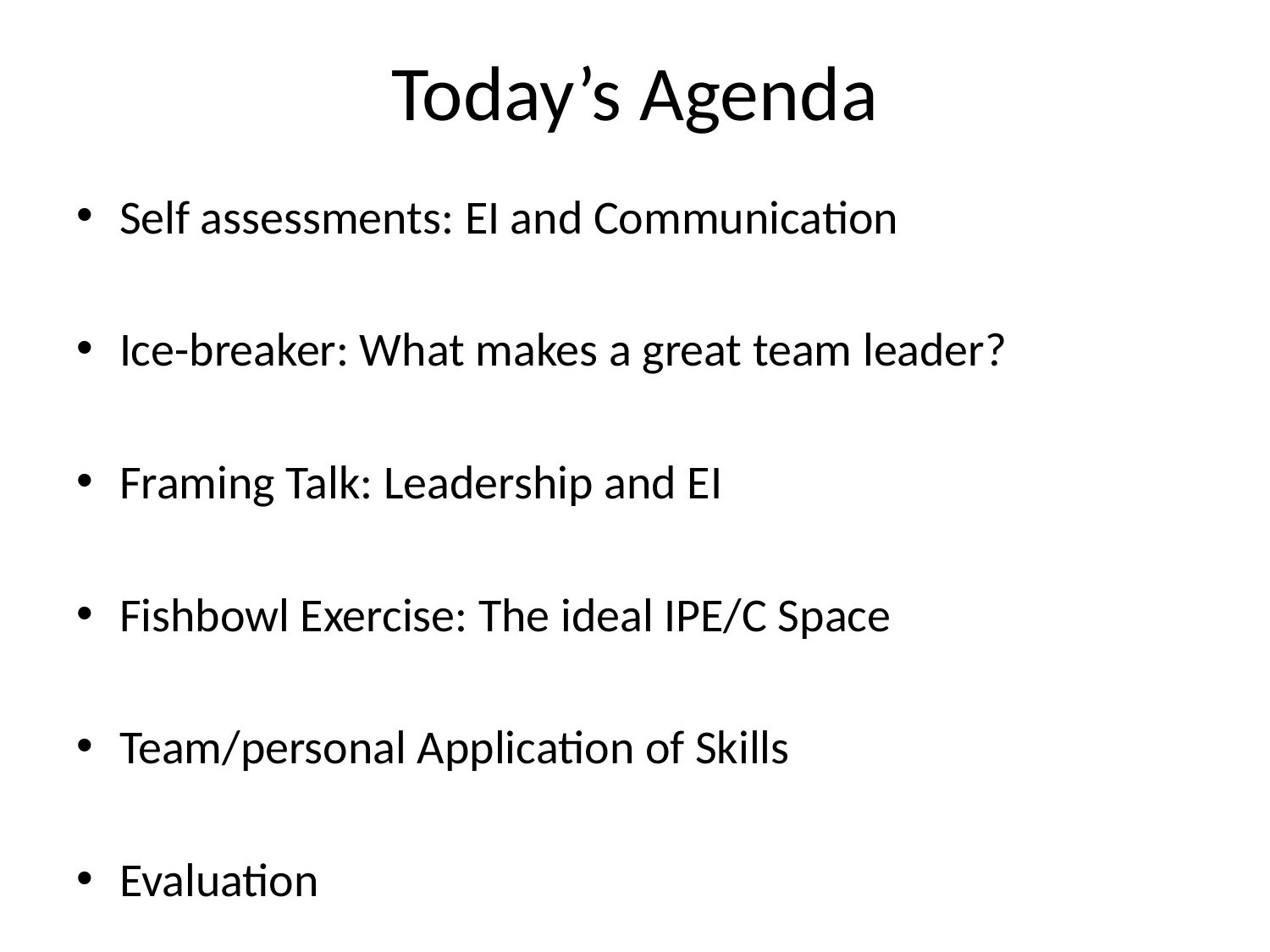

# Today’s Agenda
Self assessments: EI and Communication
Ice-breaker: What makes a great team leader?
Framing Talk: Leadership and EI
Fishbowl Exercise: The ideal IPE/C Space
Team/personal Application of Skills
Evaluation

## Slide 5
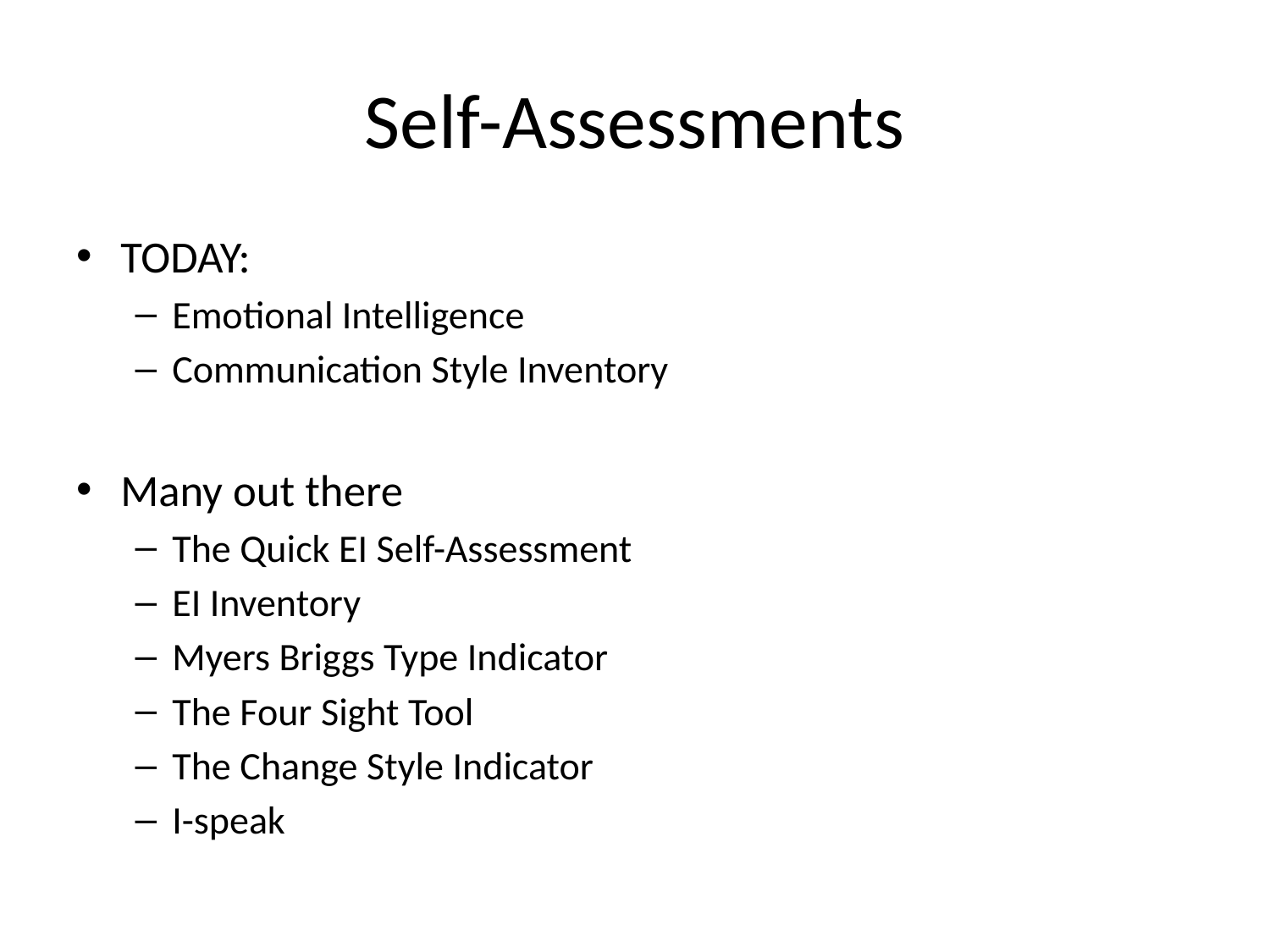

# Self-Assessments
TODAY:
Emotional Intelligence
Communication Style Inventory
Many out there
The Quick EI Self-Assessment
EI Inventory
Myers Briggs Type Indicator
The Four Sight Tool
The Change Style Indicator
I-speak

## Slide 6
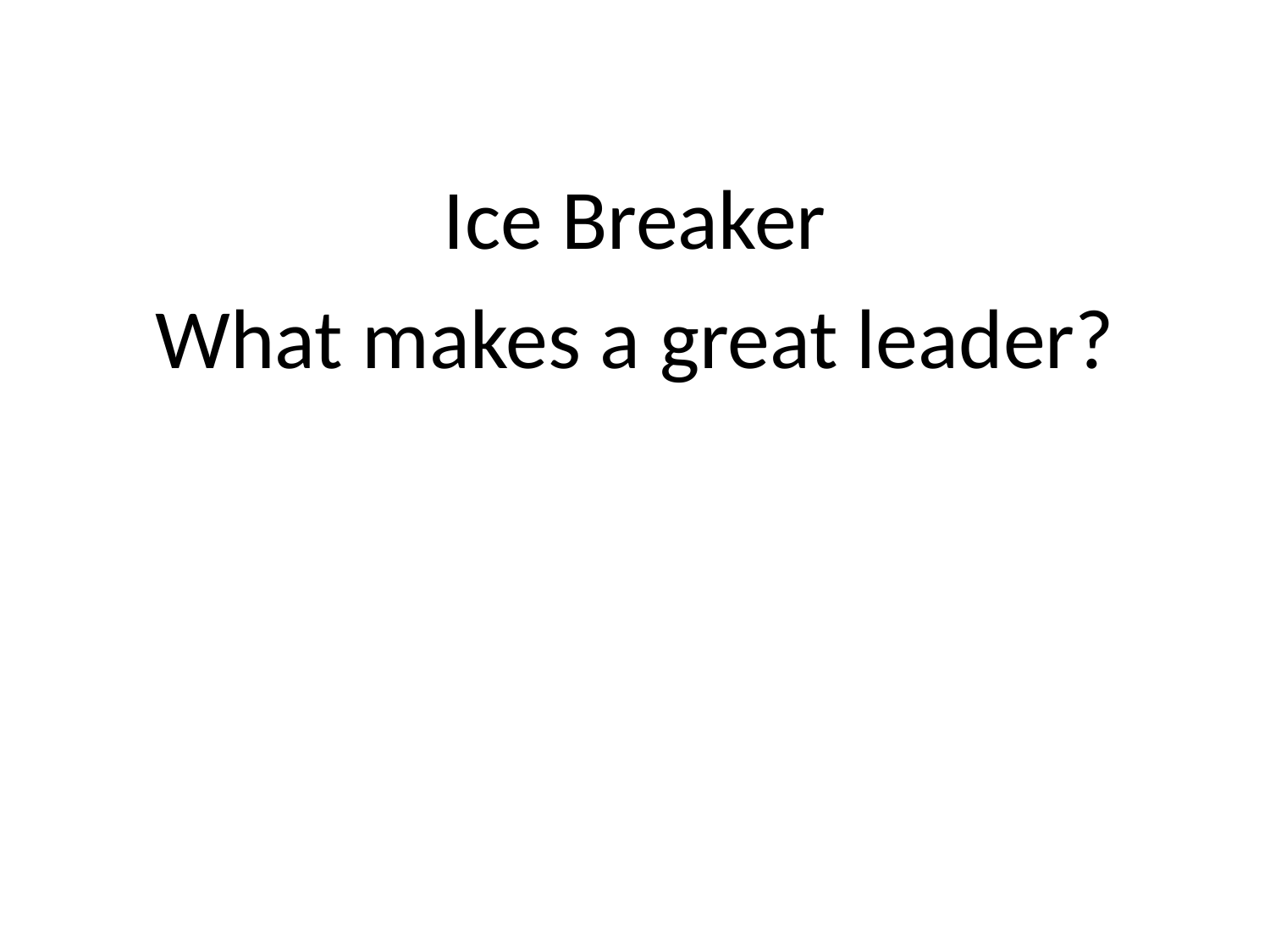

#
Ice Breaker
What makes a great leader?

## Slide 7
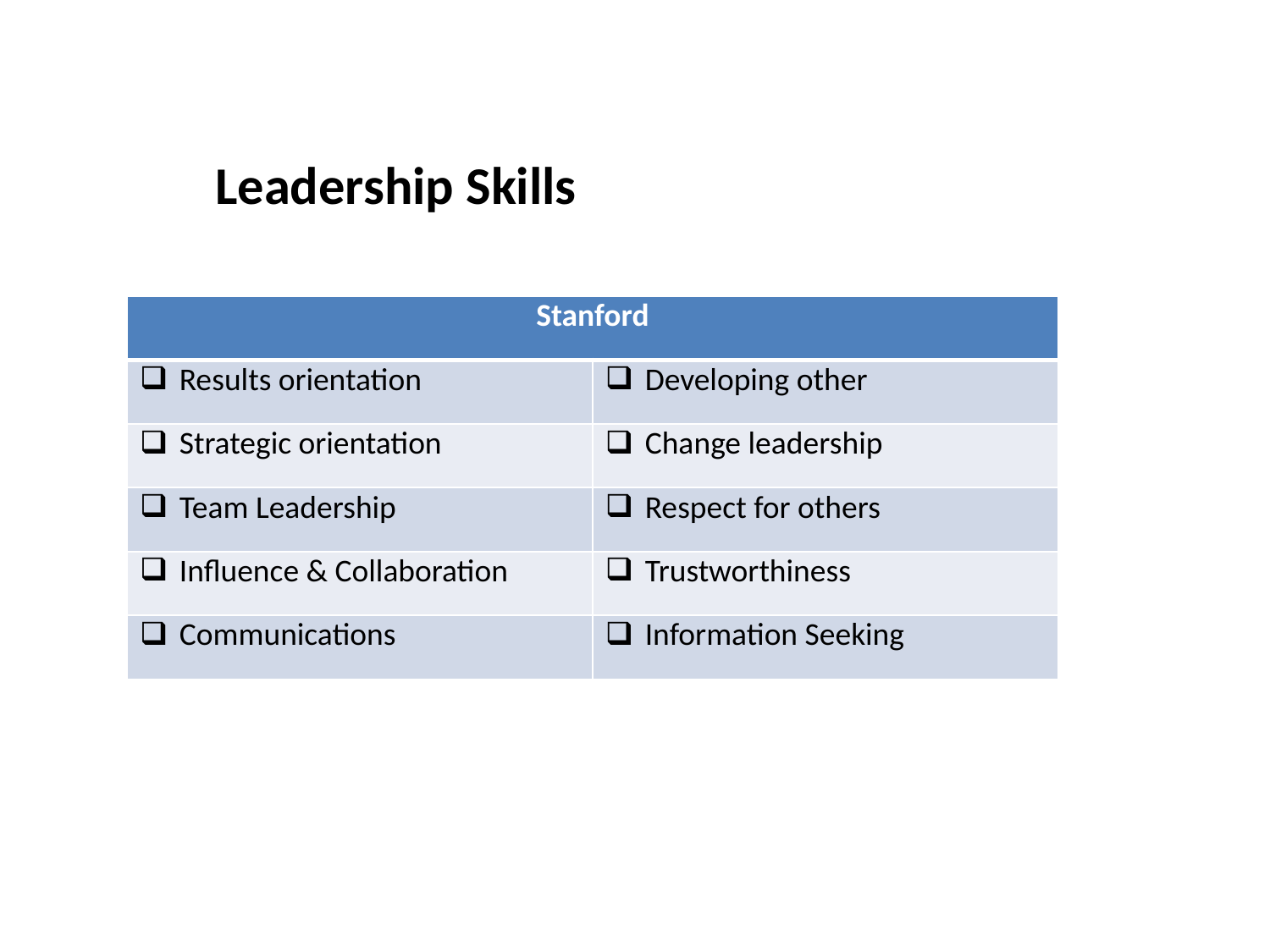

Leadership Skills
| Stanford | |
| --- | --- |
| Results orientation | Developing other |
| Strategic orientation | Change leadership |
| Team Leadership | Respect for others |
| Influence & Collaboration | Trustworthiness |
| Communications | Information Seeking |

## Slide 8
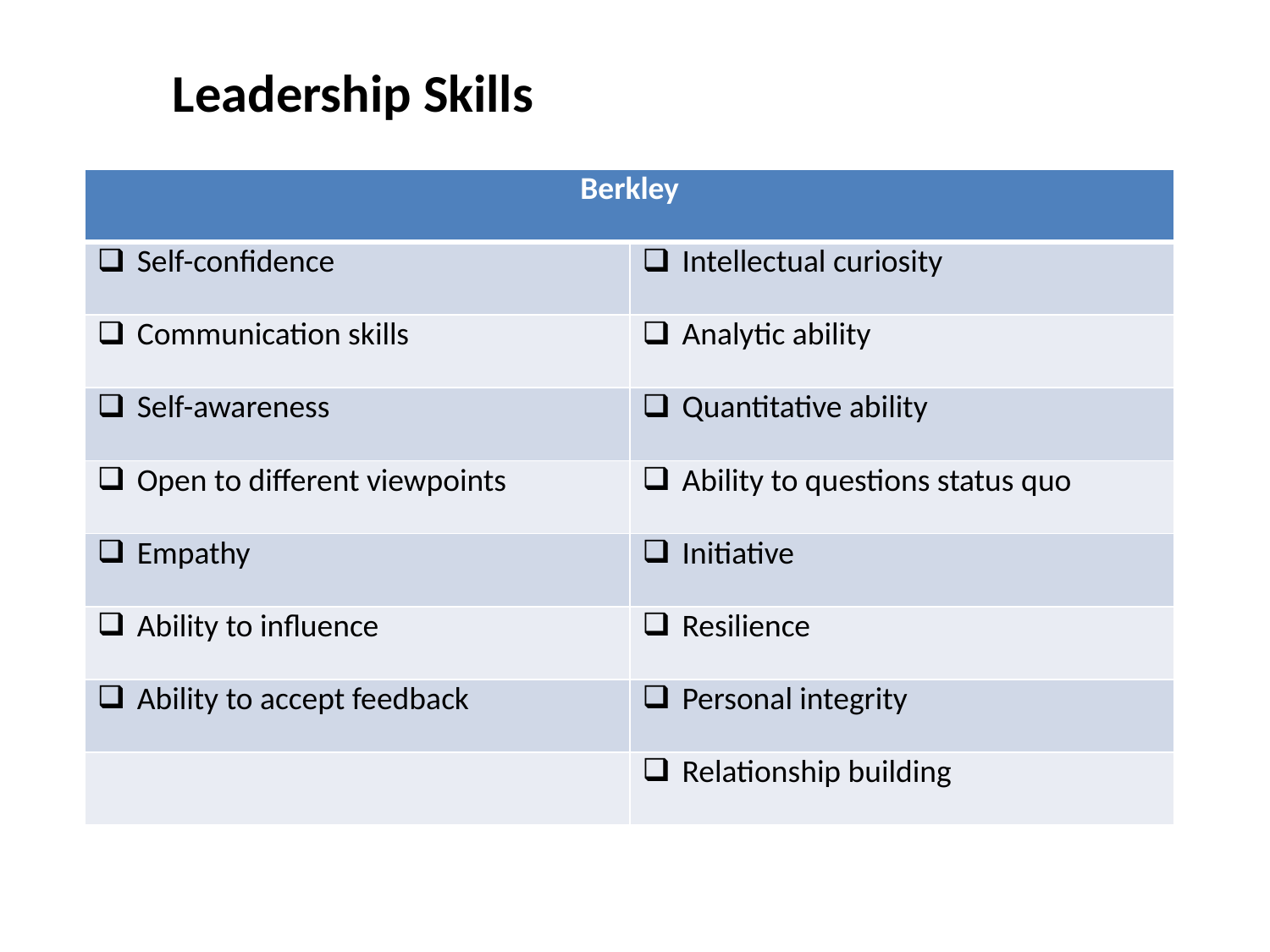

Leadership Skills
| Berkley | |
| --- | --- |
| Self-confidence | Intellectual curiosity |
| Communication skills | Analytic ability |
| Self-awareness | Quantitative ability |
| Open to different viewpoints | Ability to questions status quo |
| Empathy | Initiative |
| Ability to influence | Resilience |
| Ability to accept feedback | Personal integrity |
| | Relationship building |

## Slide 9
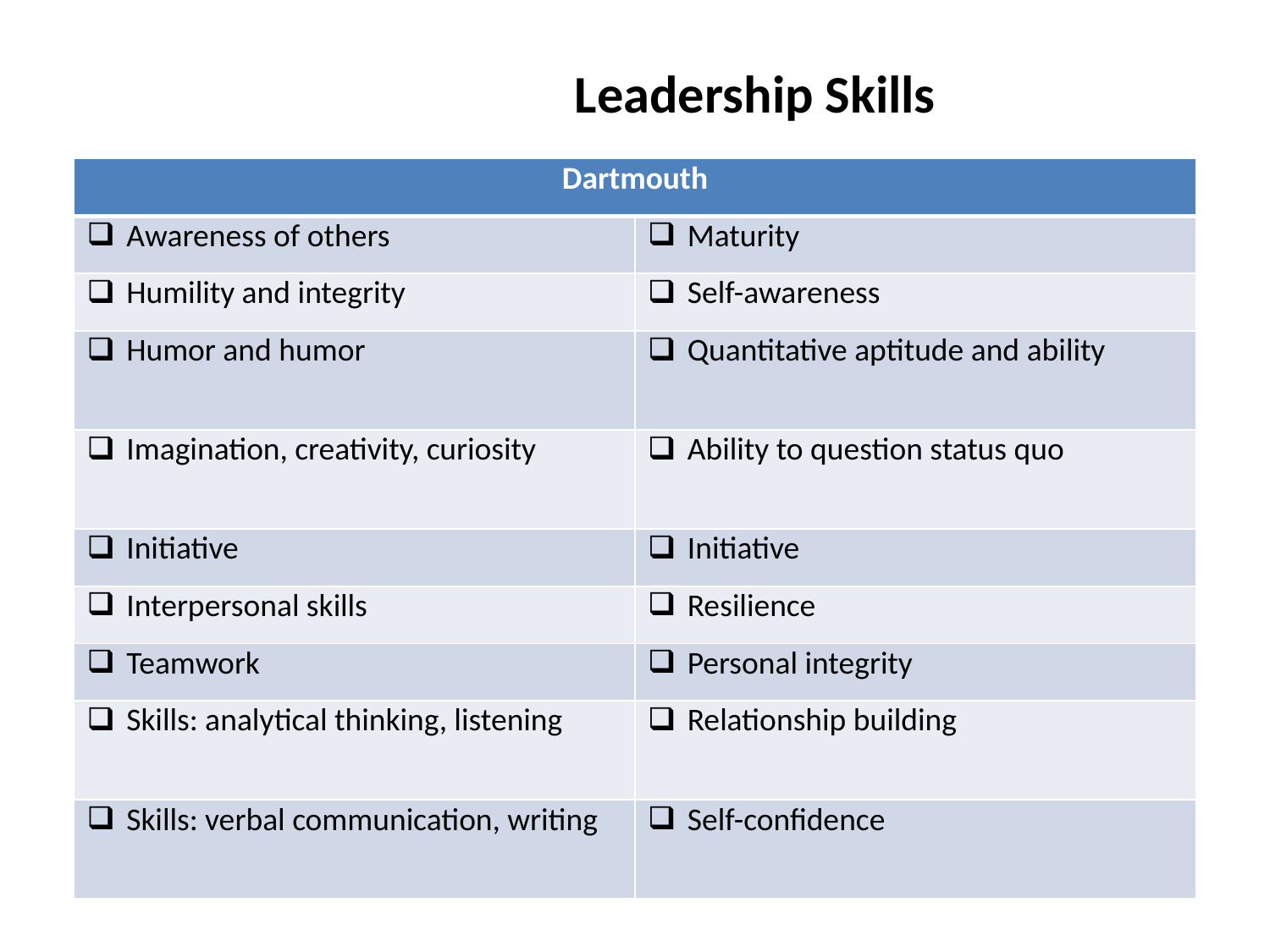

Leadership Skills
| Dartmouth | |
| --- | --- |
| Awareness of others | Maturity |
| Humility and integrity | Self-awareness |
| Humor and humor | Quantitative aptitude and ability |
| Imagination, creativity, curiosity | Ability to question status quo |
| Initiative | Initiative |
| Interpersonal skills | Resilience |
| Teamwork | Personal integrity |
| Skills: analytical thinking, listening | Relationship building |
| Skills: verbal communication, writing | Self-confidence |

## Slide 10
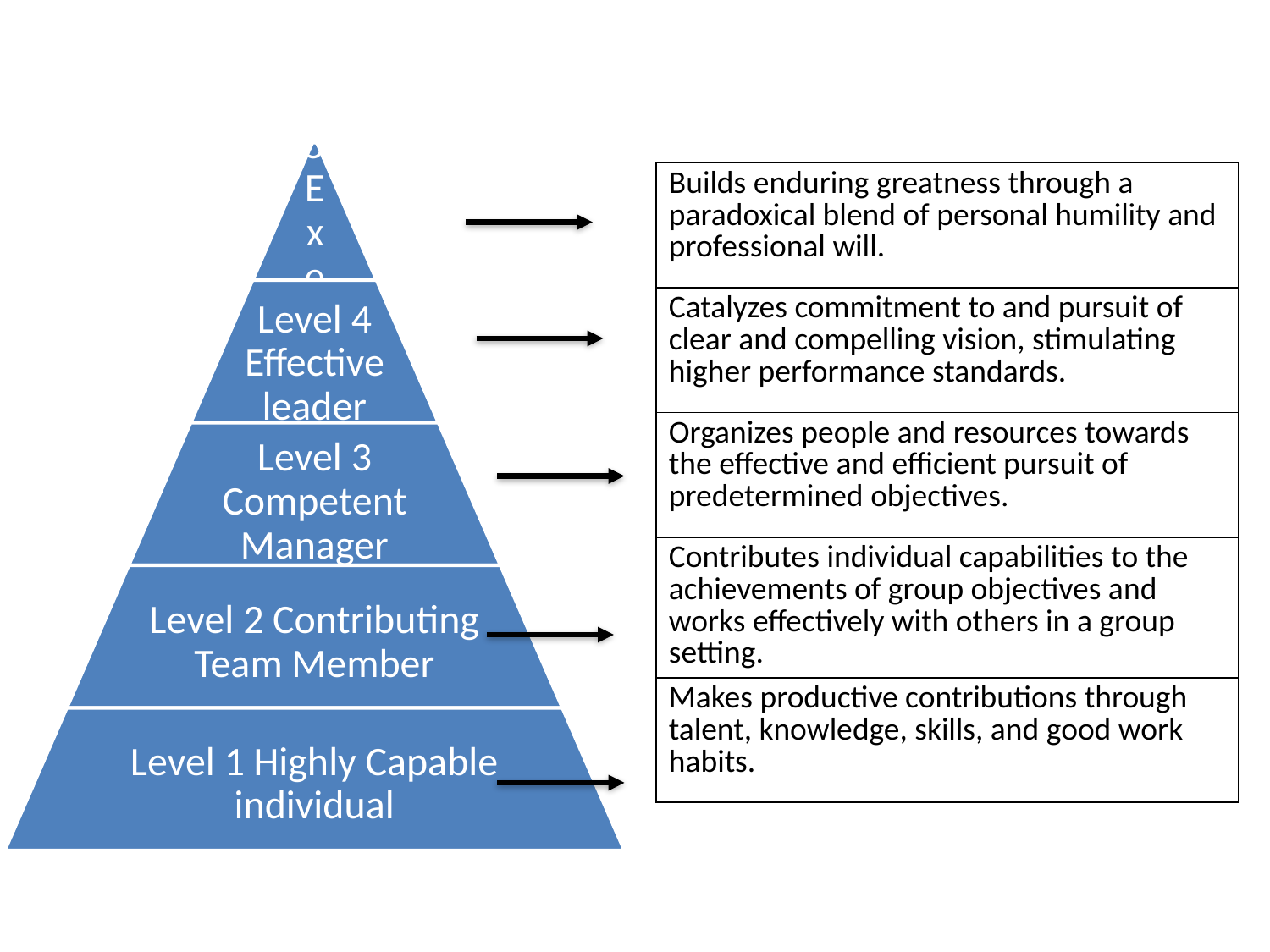

| Builds enduring greatness through a paradoxical blend of personal humility and professional will. |
| --- |
| Catalyzes commitment to and pursuit of clear and compelling vision, stimulating higher performance standards. |
| Organizes people and resources towards the effective and efficient pursuit of predetermined objectives. |
| Contributes individual capabilities to the achievements of group objectives and works effectively with others in a group setting. |
| Makes productive contributions through talent, knowledge, skills, and good work habits. |

## Slide 11
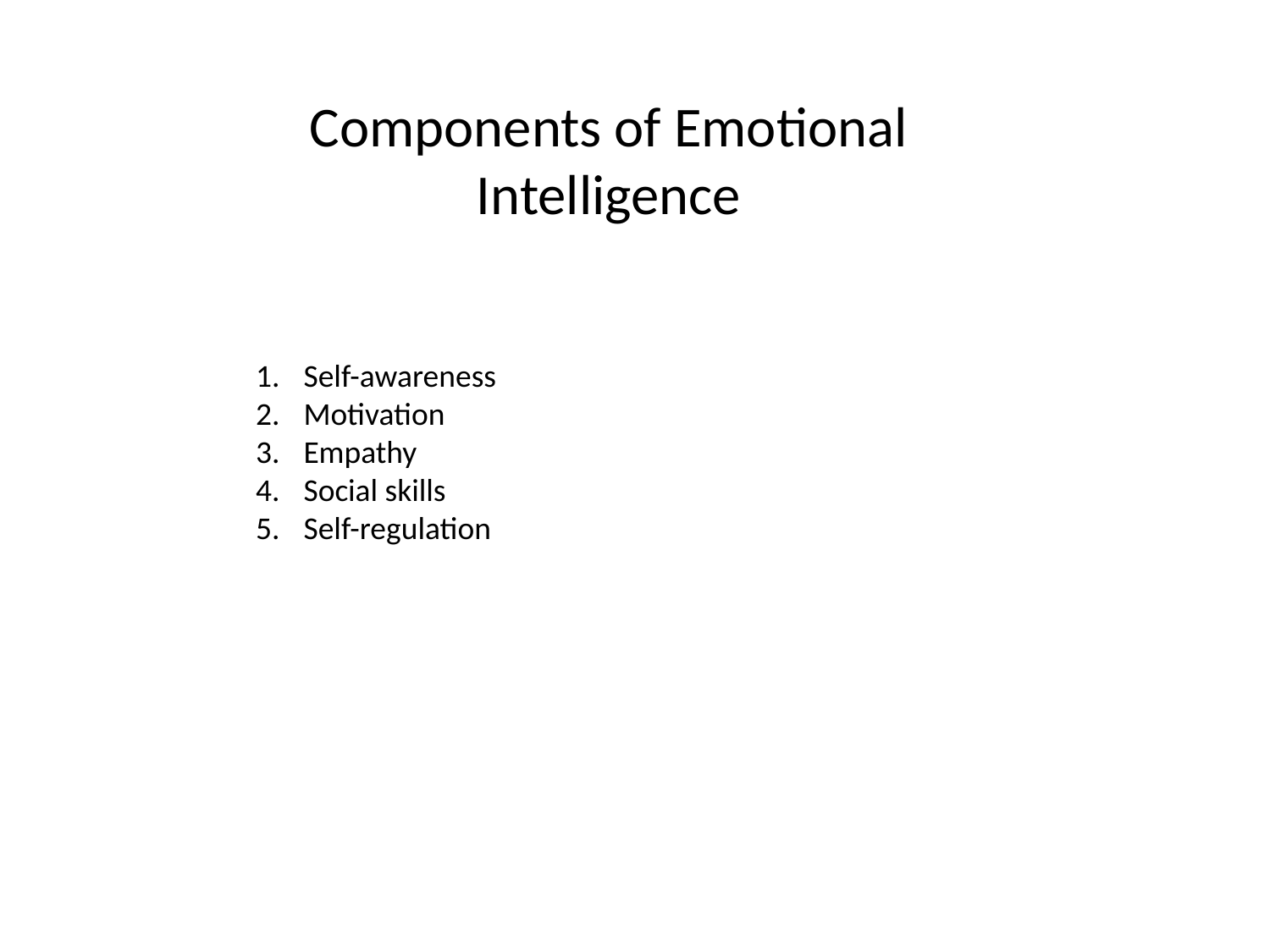

Components of Emotional Intelligence
Self-awareness
Motivation
Empathy
Social skills
Self-regulation

## Slide 12
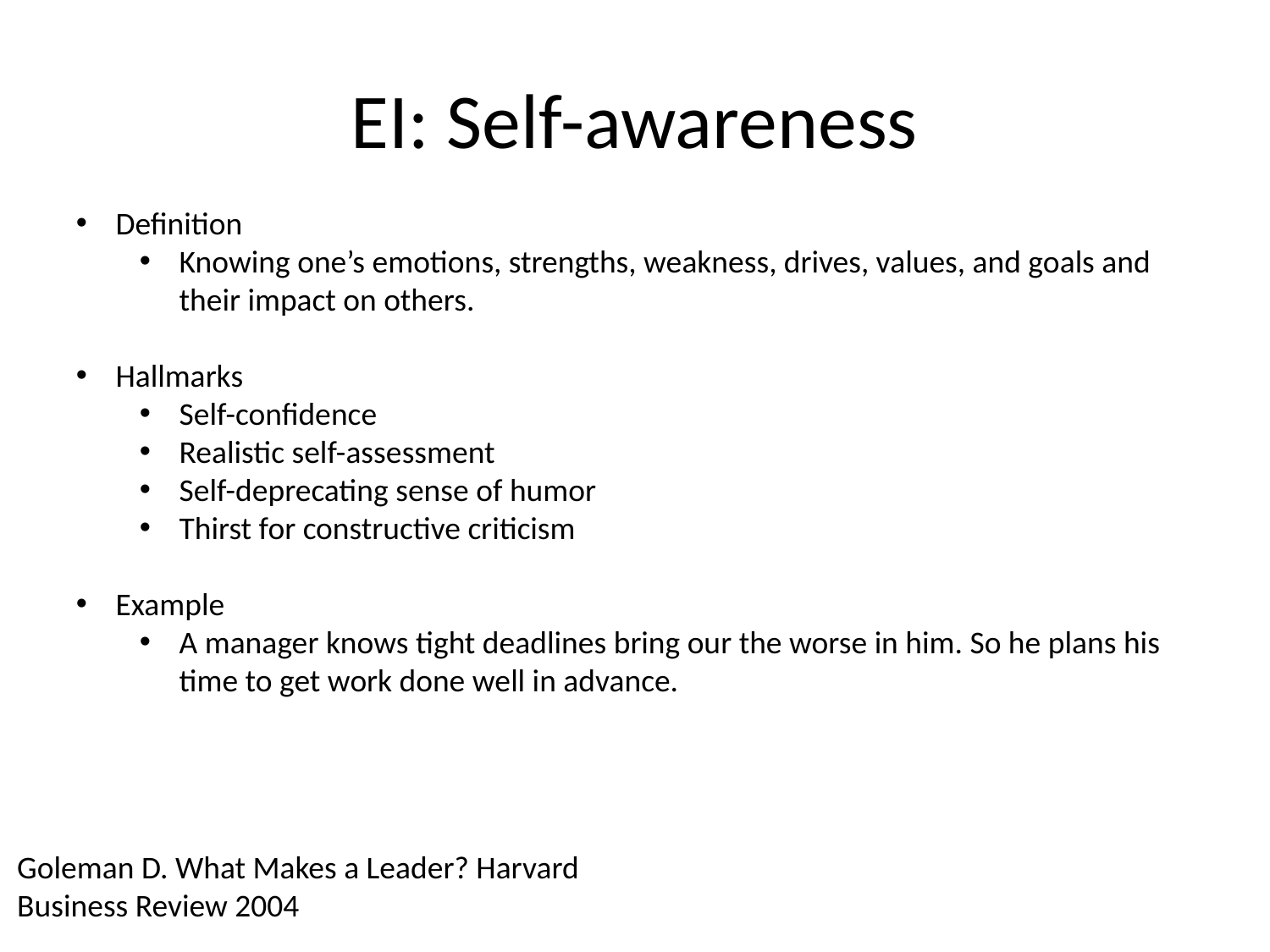

# EI: Self-awareness
Definition
Knowing one’s emotions, strengths, weakness, drives, values, and goals and their impact on others.
Hallmarks
Self-confidence
Realistic self-assessment
Self-deprecating sense of humor
Thirst for constructive criticism
Example
A manager knows tight deadlines bring our the worse in him. So he plans his time to get work done well in advance.
Goleman D. What Makes a Leader? Harvard Business Review 2004

## Slide 13
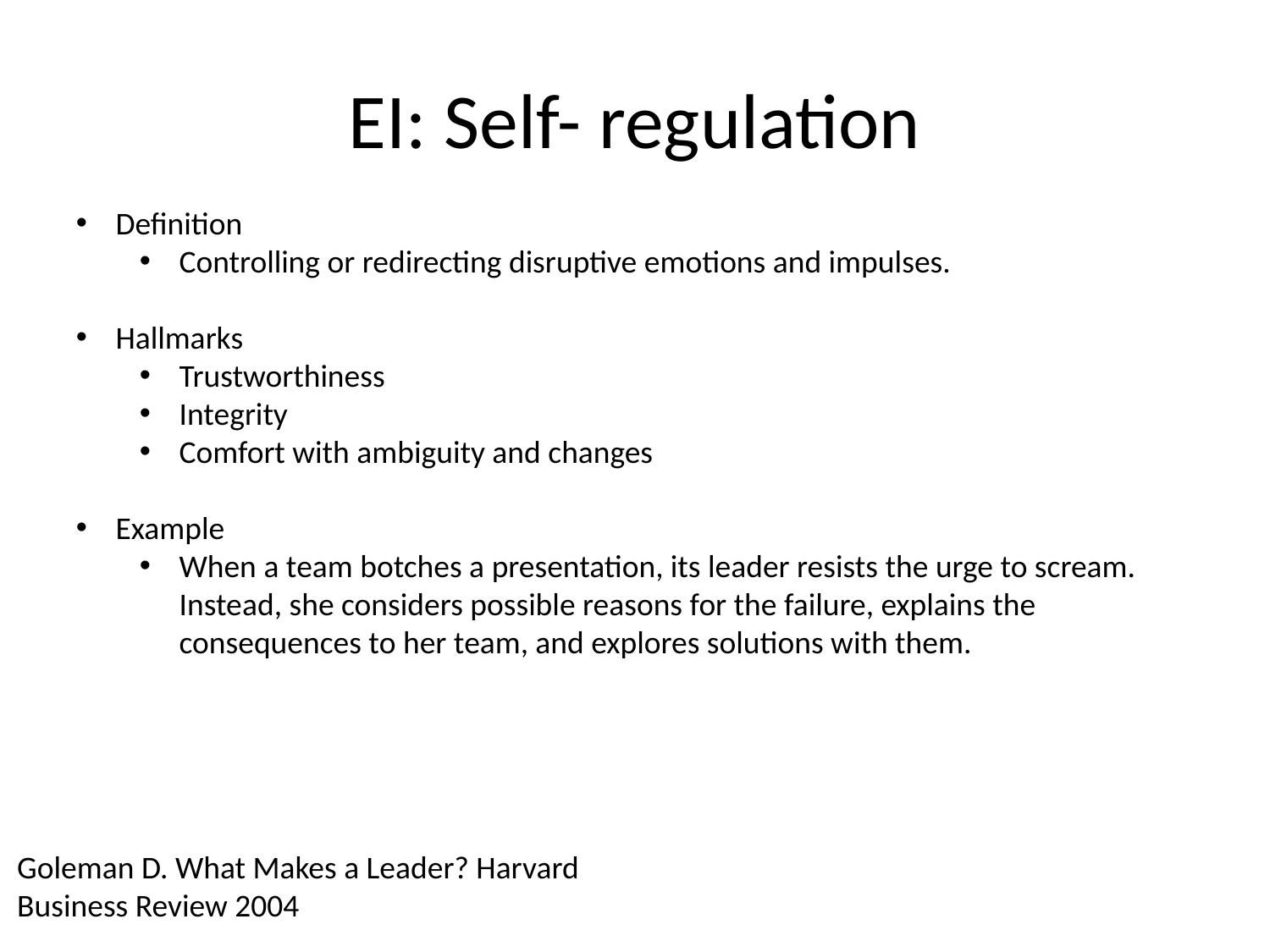

# EI: Self- regulation
Definition
Controlling or redirecting disruptive emotions and impulses.
Hallmarks
Trustworthiness
Integrity
Comfort with ambiguity and changes
Example
When a team botches a presentation, its leader resists the urge to scream. Instead, she considers possible reasons for the failure, explains the consequences to her team, and explores solutions with them.
Goleman D. What Makes a Leader? Harvard Business Review 2004

## Slide 14
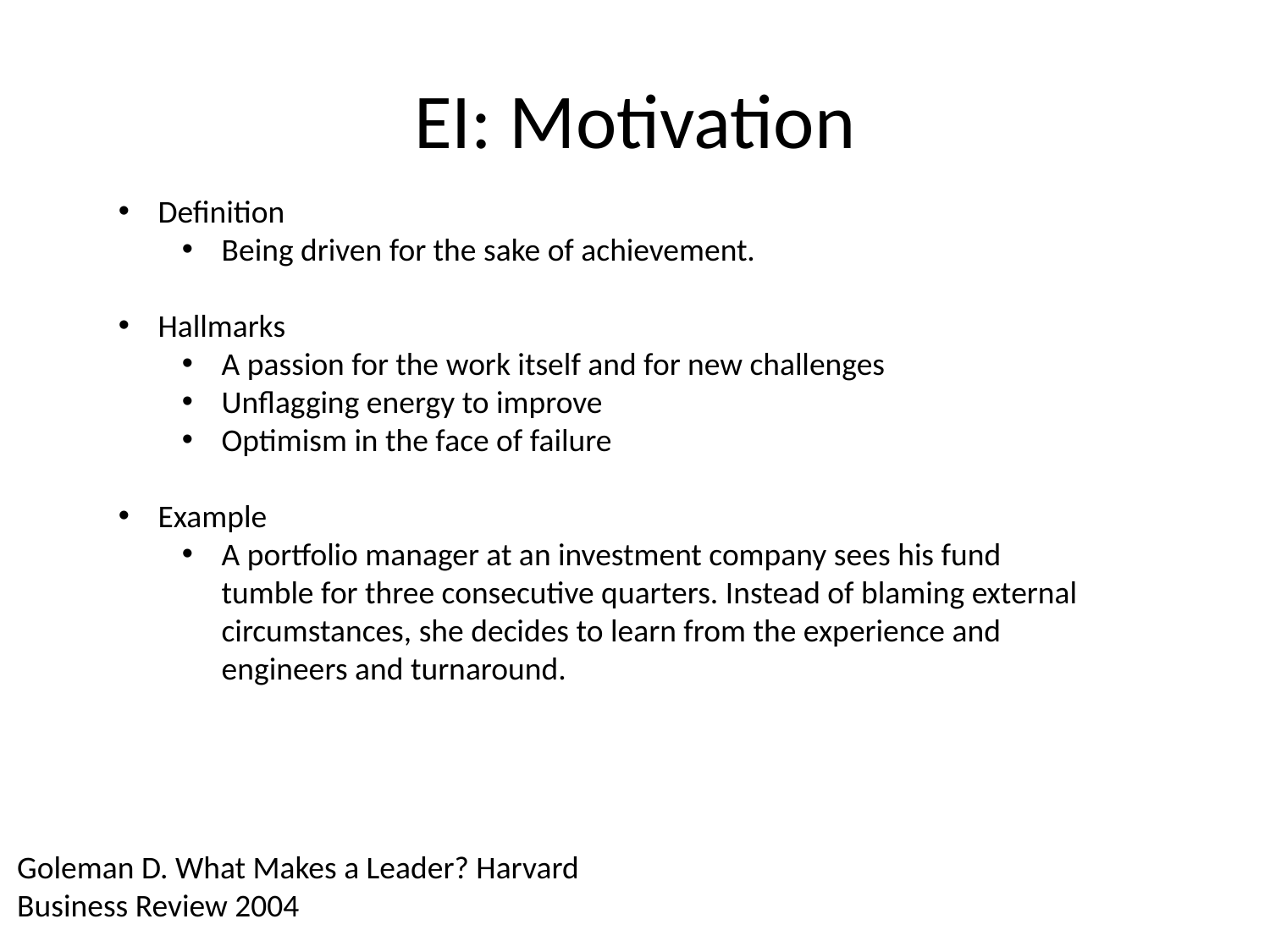

# EI: Motivation
Definition
Being driven for the sake of achievement.
Hallmarks
A passion for the work itself and for new challenges
Unflagging energy to improve
Optimism in the face of failure
Example
A portfolio manager at an investment company sees his fund tumble for three consecutive quarters. Instead of blaming external circumstances, she decides to learn from the experience and engineers and turnaround.
Goleman D. What Makes a Leader? Harvard Business Review 2004

## Slide 15
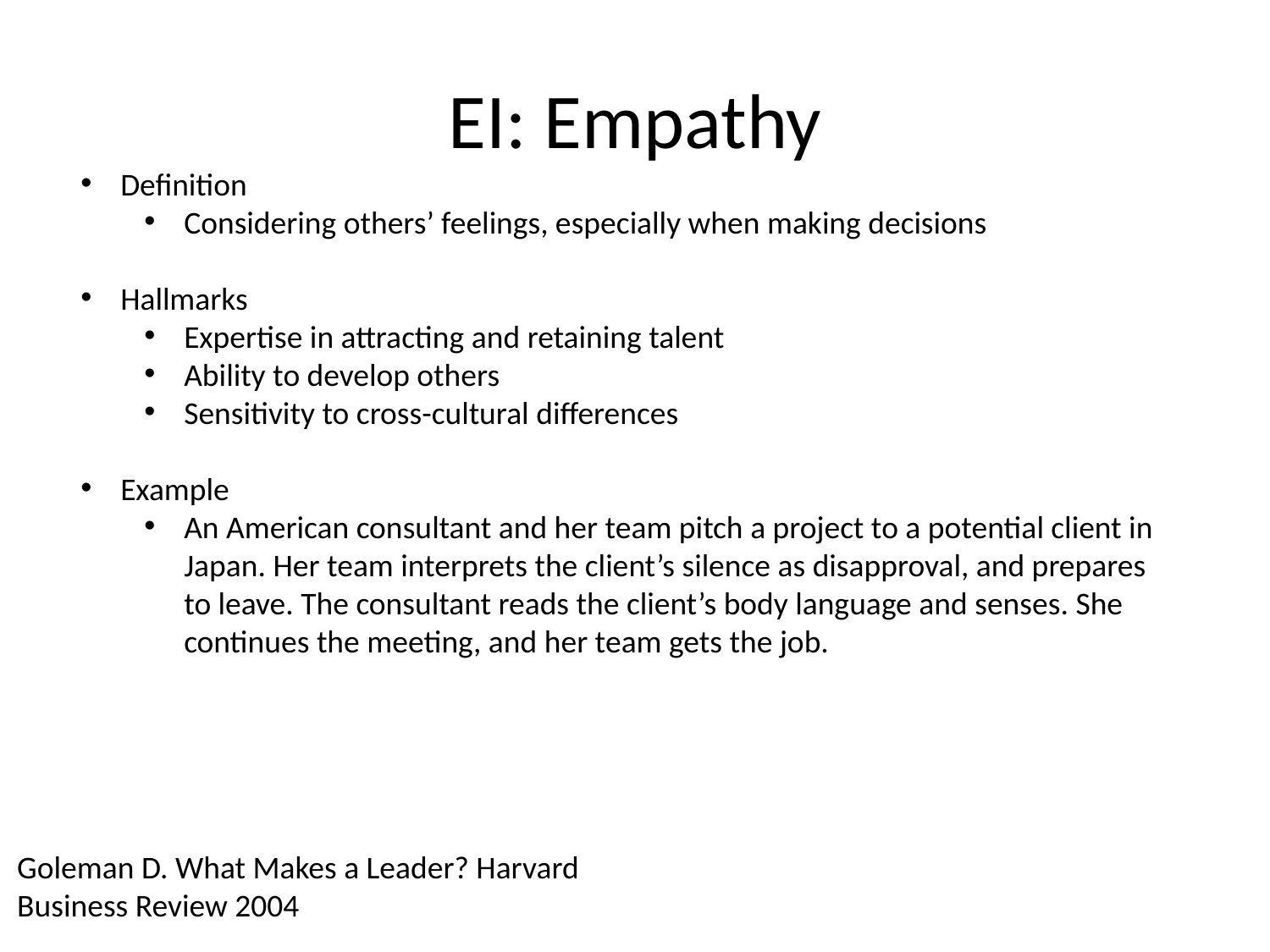

# EI: Empathy
Definition
Considering others’ feelings, especially when making decisions
Hallmarks
Expertise in attracting and retaining talent
Ability to develop others
Sensitivity to cross-cultural differences
Example
An American consultant and her team pitch a project to a potential client in Japan. Her team interprets the client’s silence as disapproval, and prepares to leave. The consultant reads the client’s body language and senses. She continues the meeting, and her team gets the job.
Goleman D. What Makes a Leader? Harvard Business Review 2004

## Slide 16
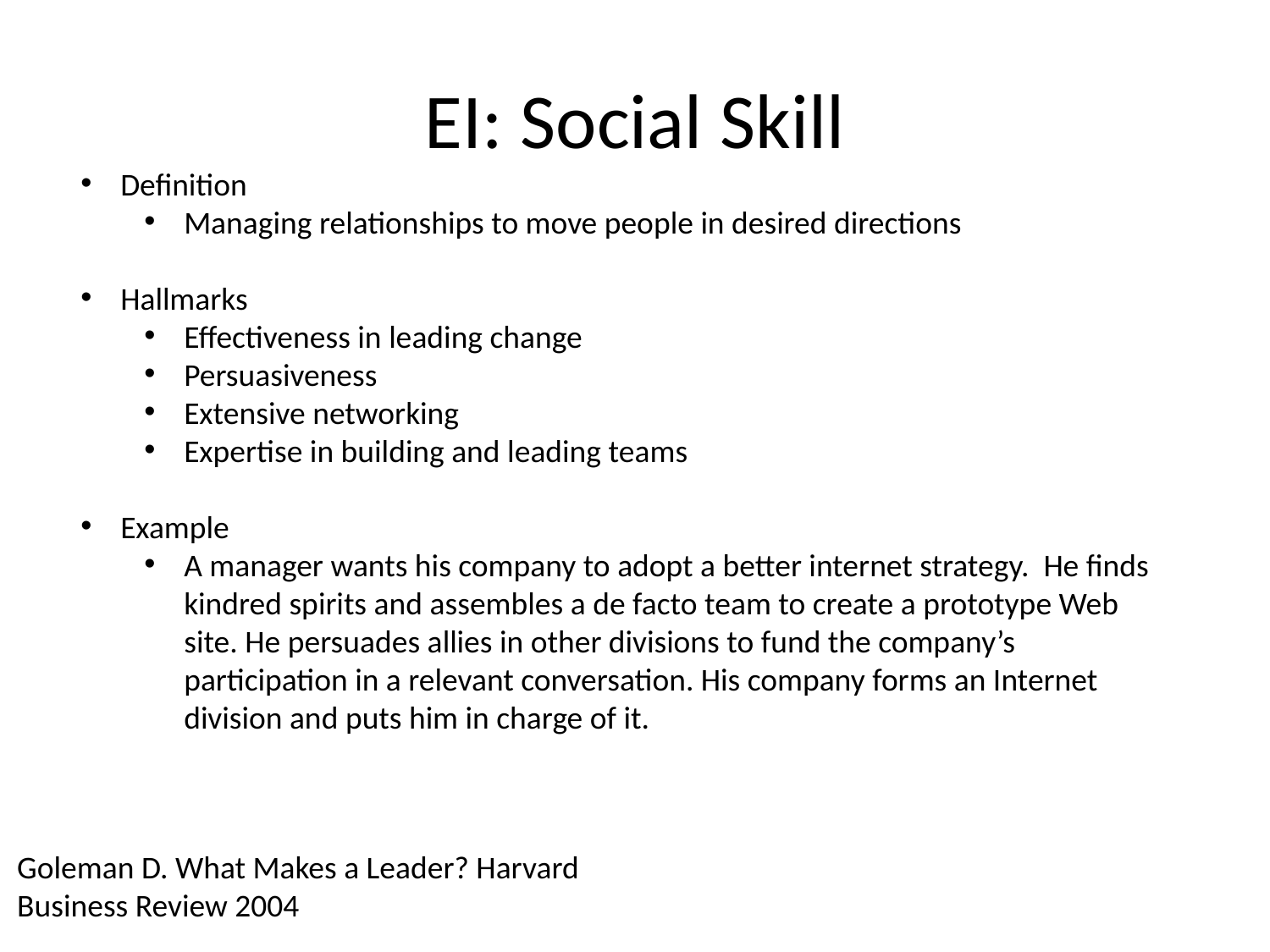

# EI: Social Skill
Definition
Managing relationships to move people in desired directions
Hallmarks
Effectiveness in leading change
Persuasiveness
Extensive networking
Expertise in building and leading teams
Example
A manager wants his company to adopt a better internet strategy. He finds kindred spirits and assembles a de facto team to create a prototype Web site. He persuades allies in other divisions to fund the company’s participation in a relevant conversation. His company forms an Internet division and puts him in charge of it.
Goleman D. What Makes a Leader? Harvard Business Review 2004

## Slide 17
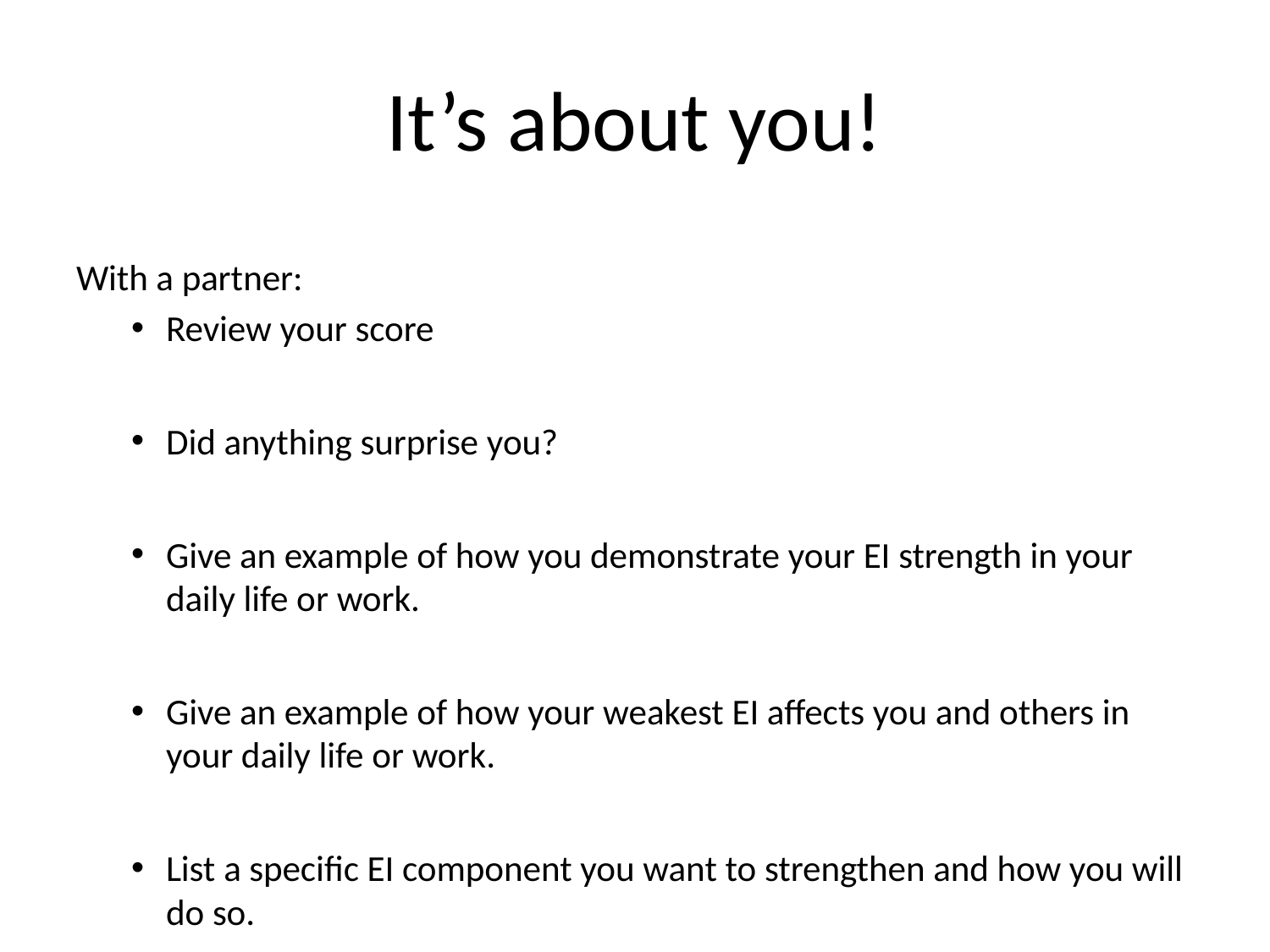

# It’s about you!
With a partner:
Review your score
Did anything surprise you?
Give an example of how you demonstrate your EI strength in your daily life or work.
Give an example of how your weakest EI affects you and others in your daily life or work.
List a specific EI component you want to strengthen and how you will do so.

## Slide 18
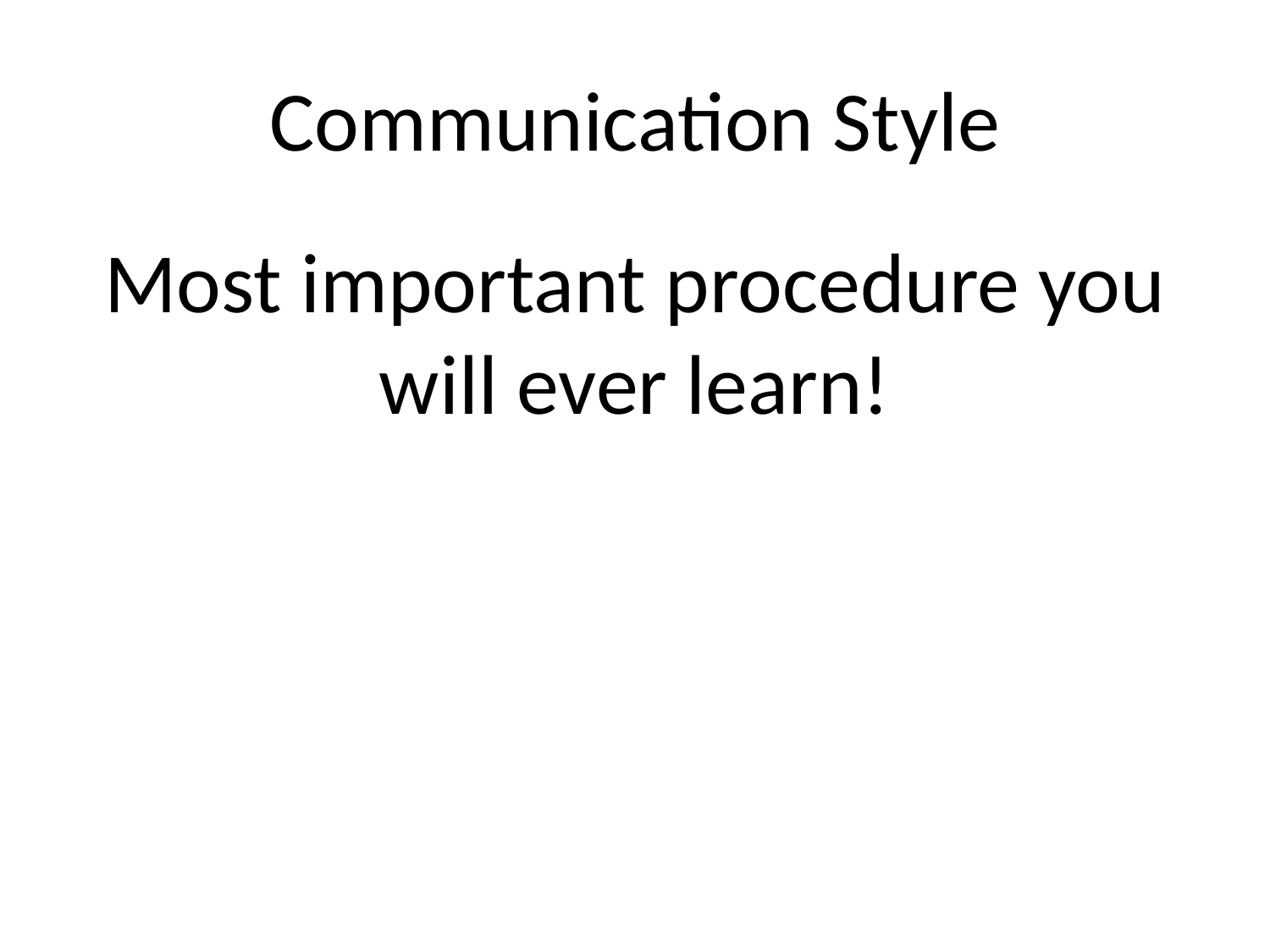

# Communication Style
Most important procedure you will ever learn!

## Slide 19
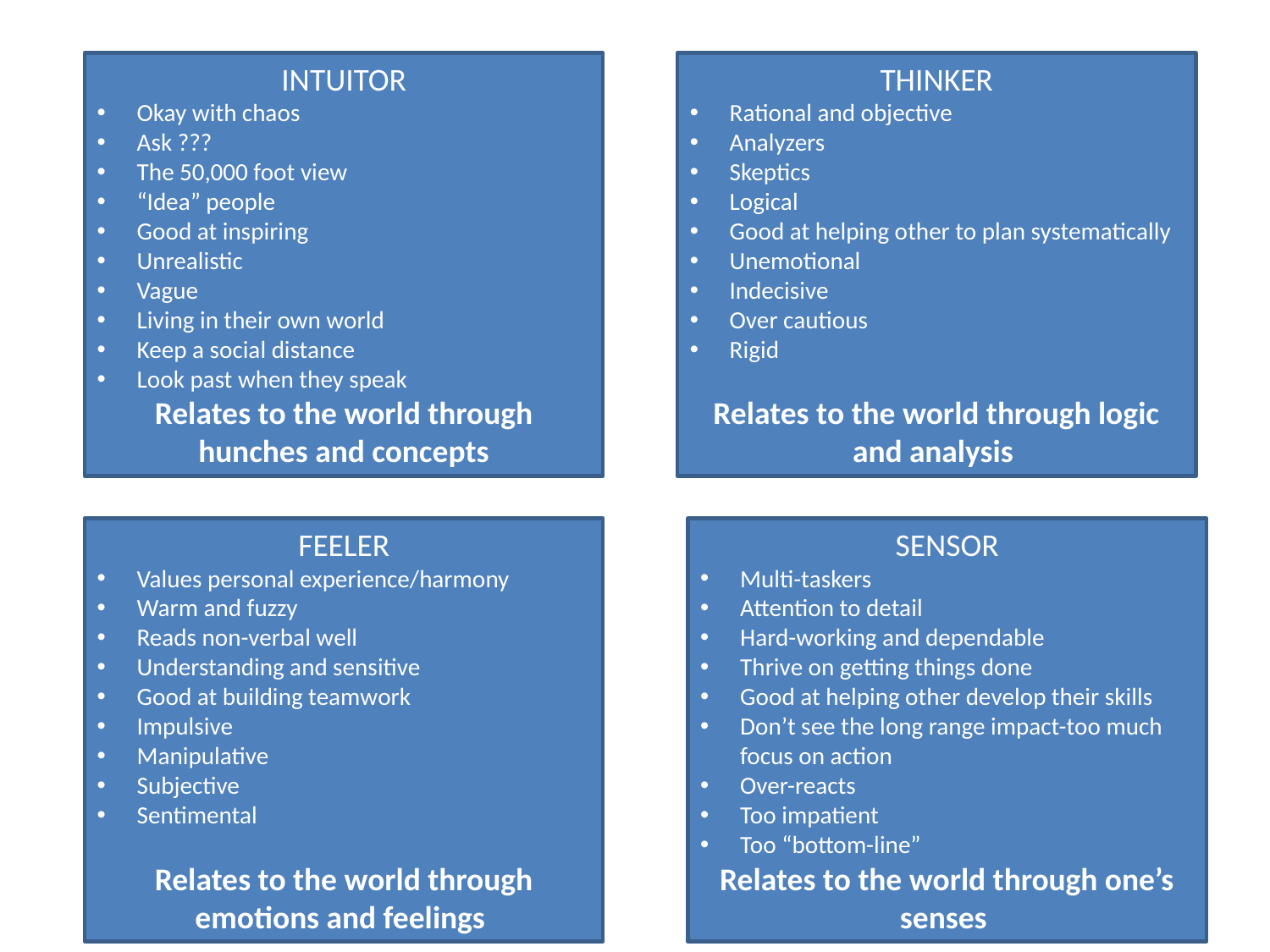

INTUITOR
Okay with chaos
Ask ???
The 50,000 foot view
“Idea” people
Good at inspiring
Unrealistic
Vague
Living in their own world
Keep a social distance
Look past when they speak
Relates to the world through hunches and concepts
THINKER
Rational and objective
Analyzers
Skeptics
Logical
Good at helping other to plan systematically
Unemotional
Indecisive
Over cautious
Rigid
Relates to the world through logic and analysis
FEELER
Values personal experience/harmony
Warm and fuzzy
Reads non-verbal well
Understanding and sensitive
Good at building teamwork
Impulsive
Manipulative
Subjective
Sentimental
Relates to the world through emotions and feelings
SENSOR
Multi-taskers
Attention to detail
Hard-working and dependable
Thrive on getting things done
Good at helping other develop their skills
Don’t see the long range impact-too much focus on action
Over-reacts
Too impatient
Too “bottom-line”
Relates to the world through one’s senses

## Slide 20
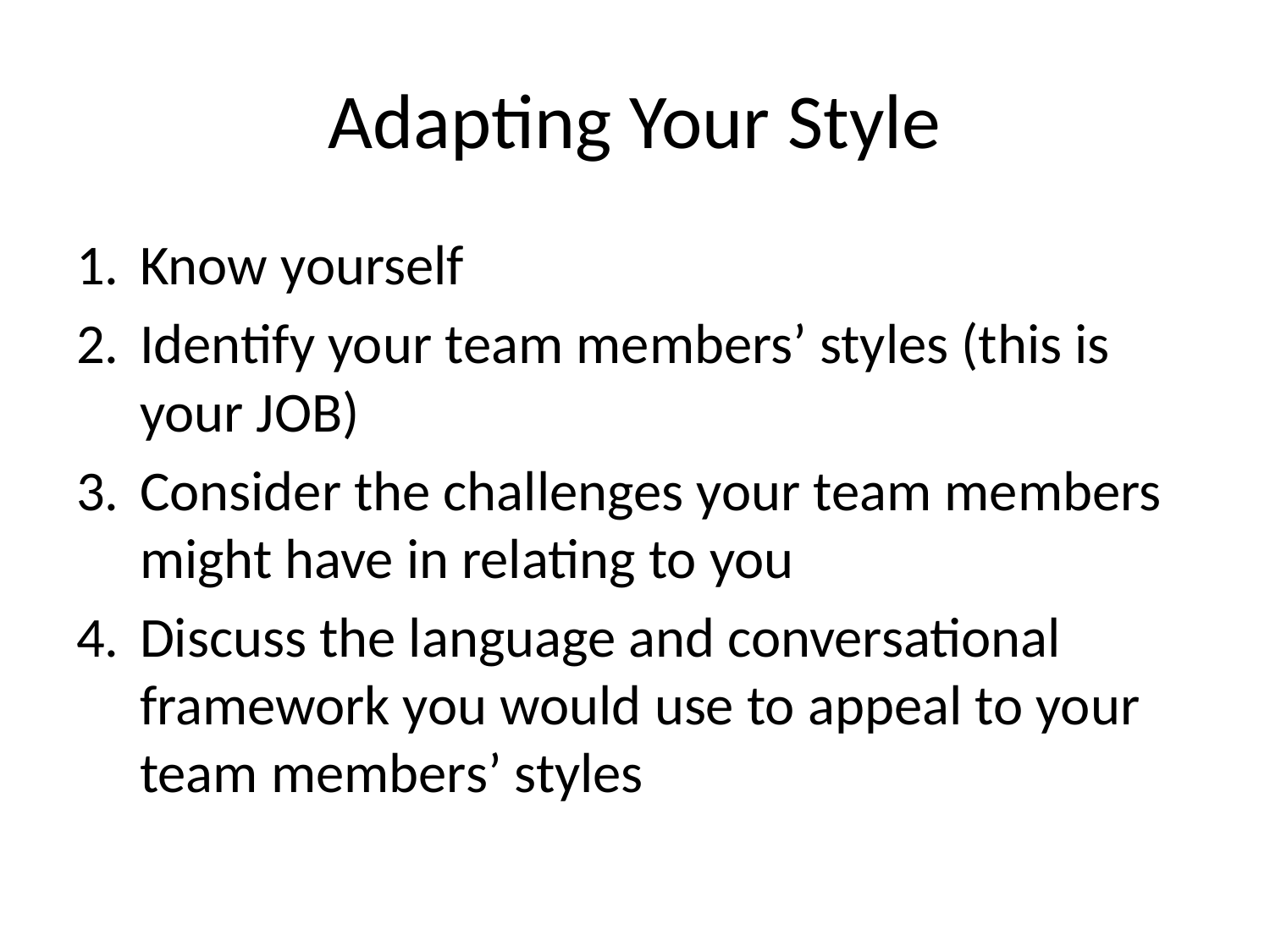

# Adapting Your Style
Know yourself
Identify your team members’ styles (this is your JOB)
Consider the challenges your team members might have in relating to you
Discuss the language and conversational framework you would use to appeal to your team members’ styles

## Slide 21
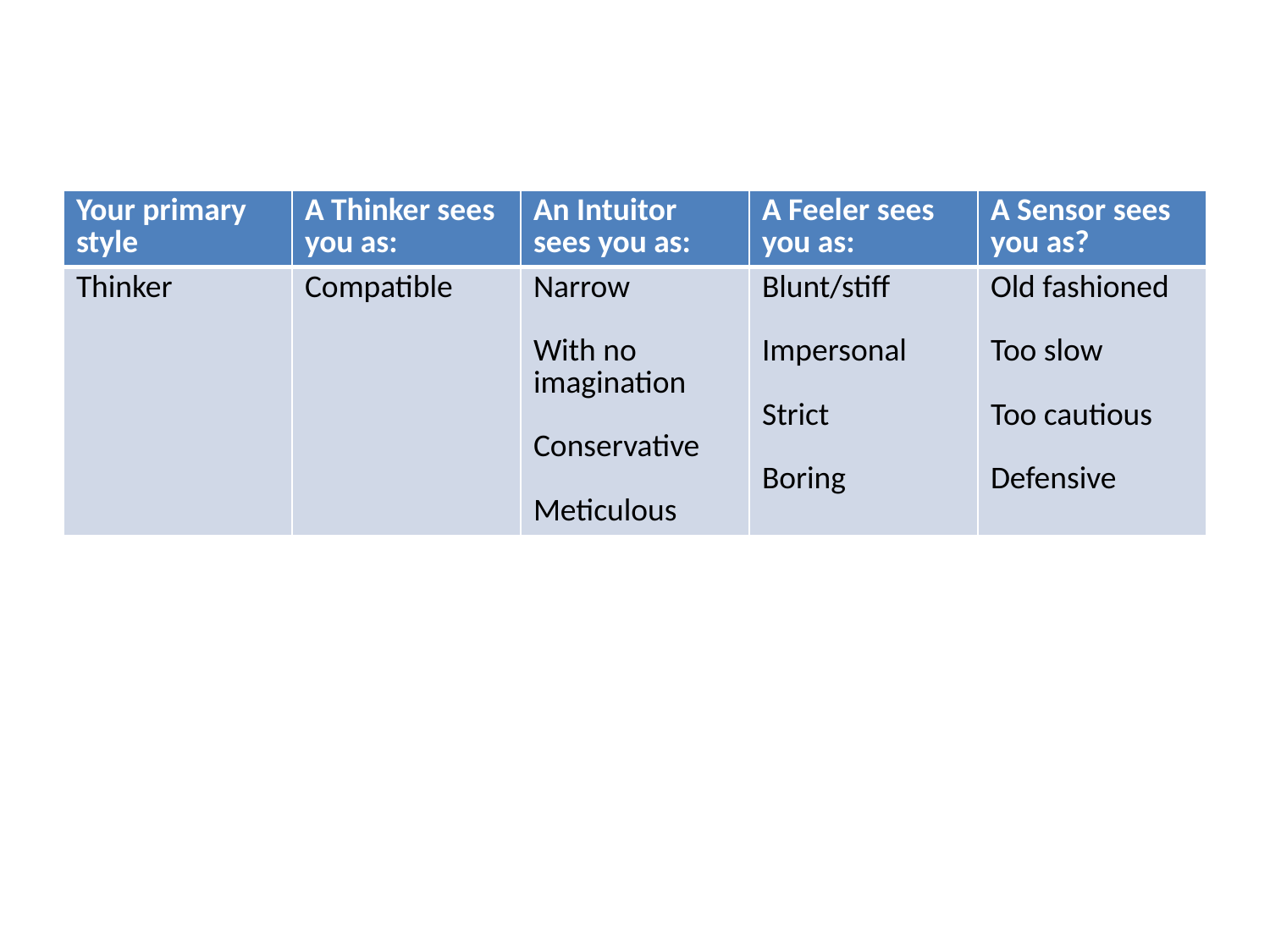

| Your primary style | A Thinker sees you as: | An Intuitor sees you as: | A Feeler sees you as: | A Sensor sees you as? |
| --- | --- | --- | --- | --- |
| Thinker | Compatible | Narrow With no imagination Conservative Meticulous | Blunt/stiff Impersonal Strict Boring | Old fashioned Too slow Too cautious Defensive |

## Slide 22
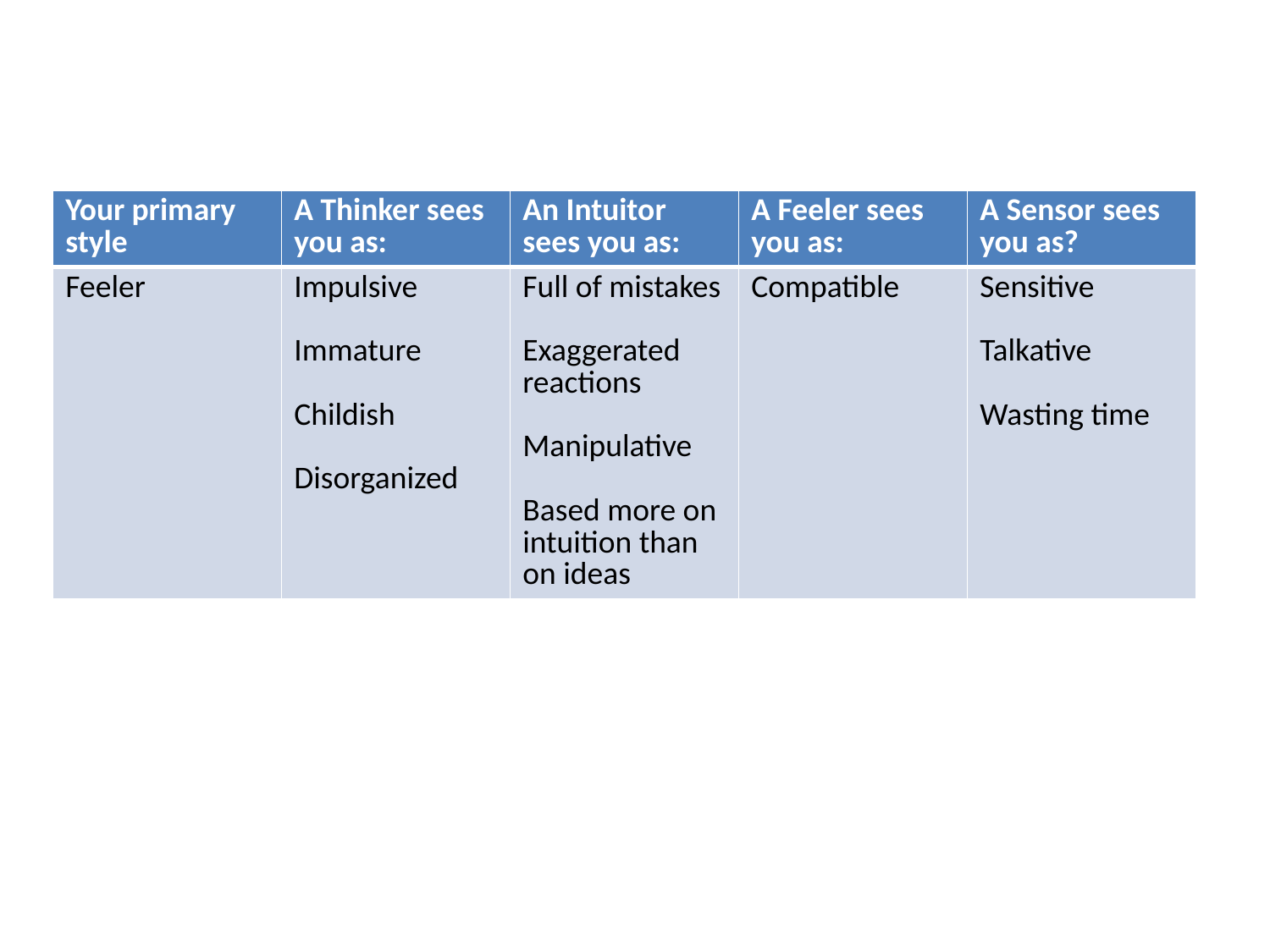

| Your primary style | A Thinker sees you as: | An Intuitor sees you as: | A Feeler sees you as: | A Sensor sees you as? |
| --- | --- | --- | --- | --- |
| Feeler | Impulsive Immature Childish Disorganized | Full of mistakes Exaggerated reactions Manipulative Based more on intuition than on ideas | Compatible | Sensitive Talkative Wasting time |

## Slide 23
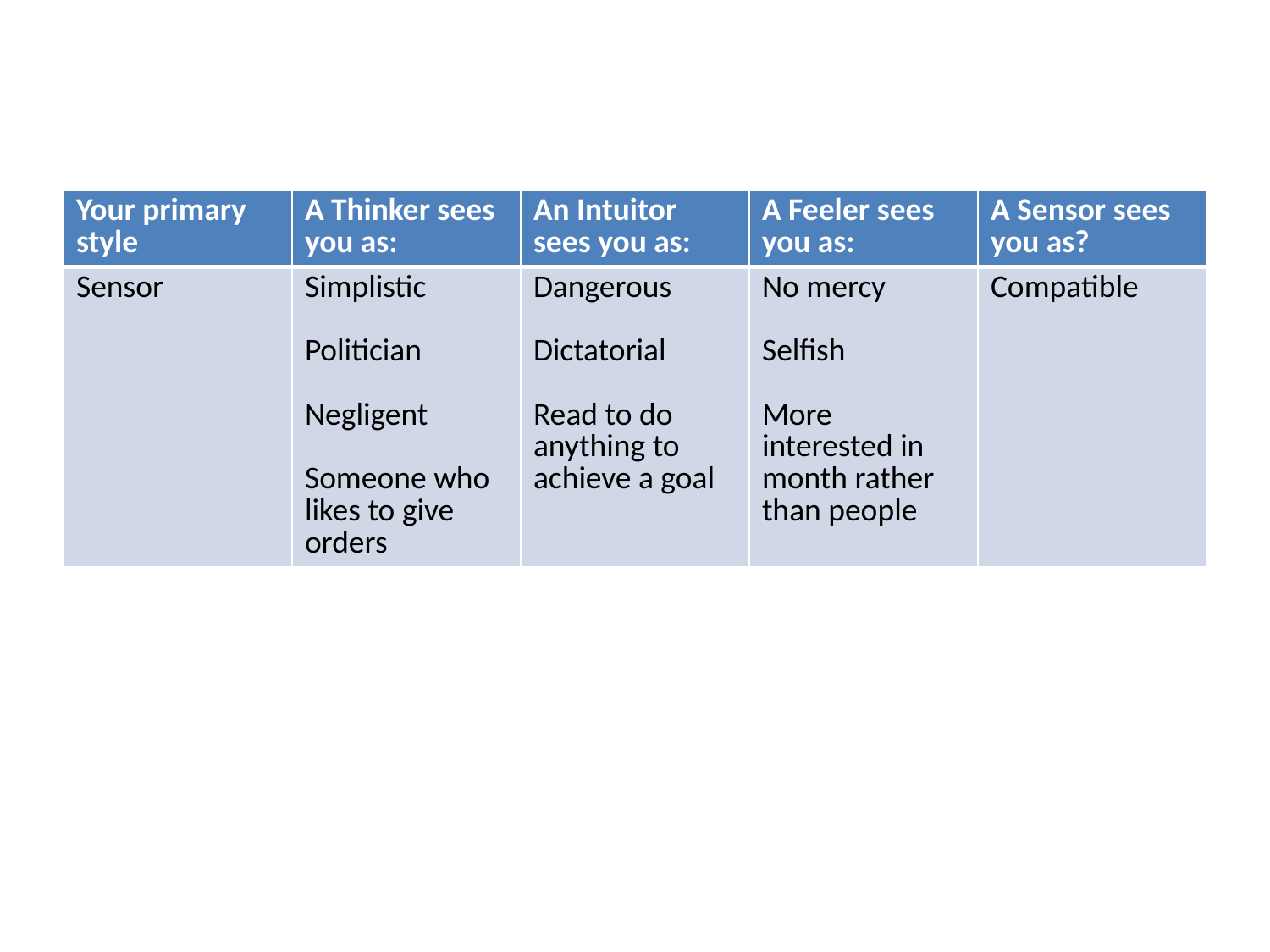

| Your primary style | A Thinker sees you as: | An Intuitor sees you as: | A Feeler sees you as: | A Sensor sees you as? |
| --- | --- | --- | --- | --- |
| Sensor | Simplistic Politician Negligent Someone who likes to give orders | Dangerous Dictatorial Read to do anything to achieve a goal | No mercy Selfish More interested in month rather than people | Compatible |

## Slide 24
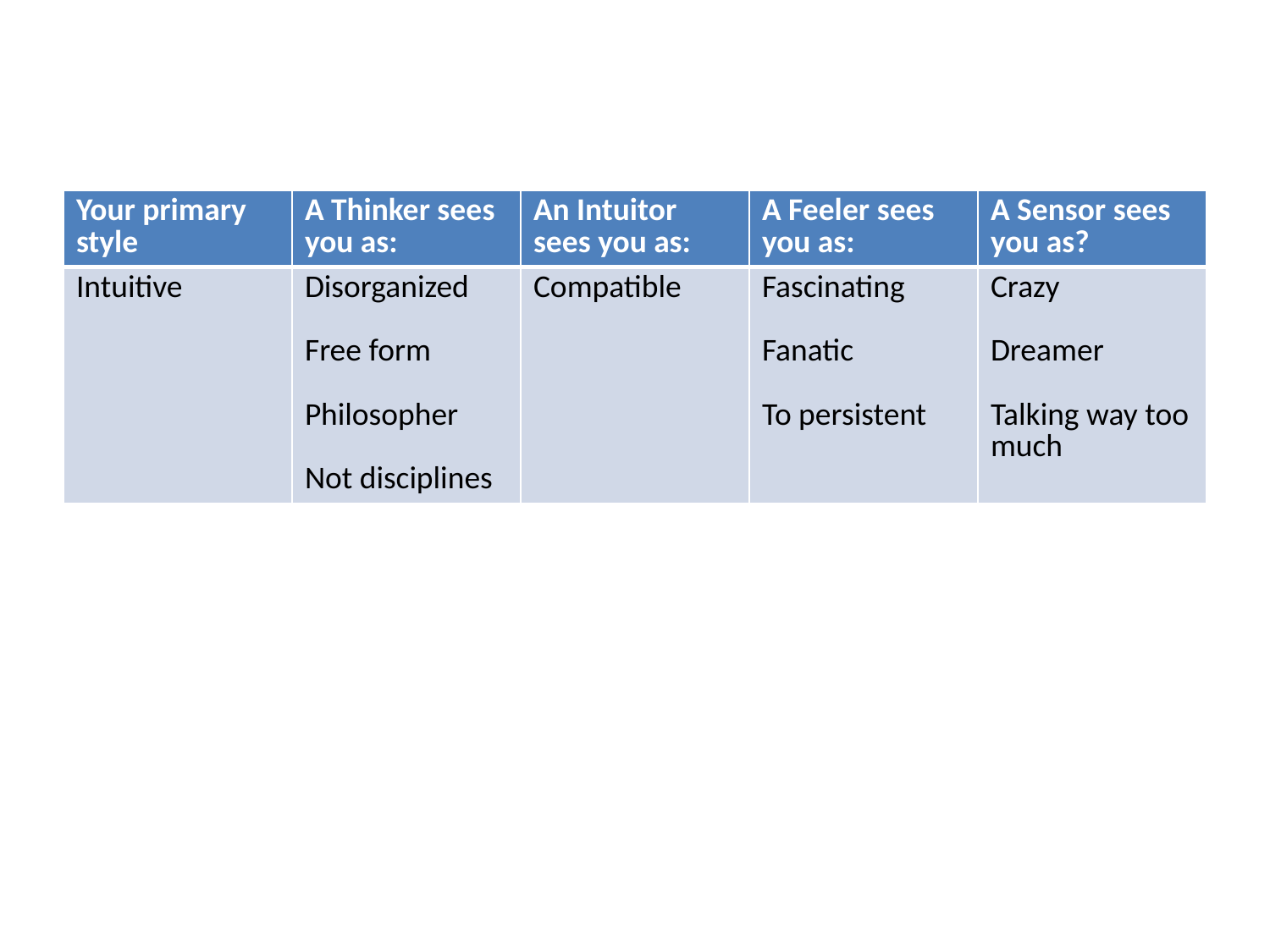

| Your primary style | A Thinker sees you as: | An Intuitor sees you as: | A Feeler sees you as: | A Sensor sees you as? |
| --- | --- | --- | --- | --- |
| Intuitive | Disorganized Free form Philosopher Not disciplines | Compatible | Fascinating Fanatic To persistent | Crazy Dreamer Talking way too much |

## Slide 25
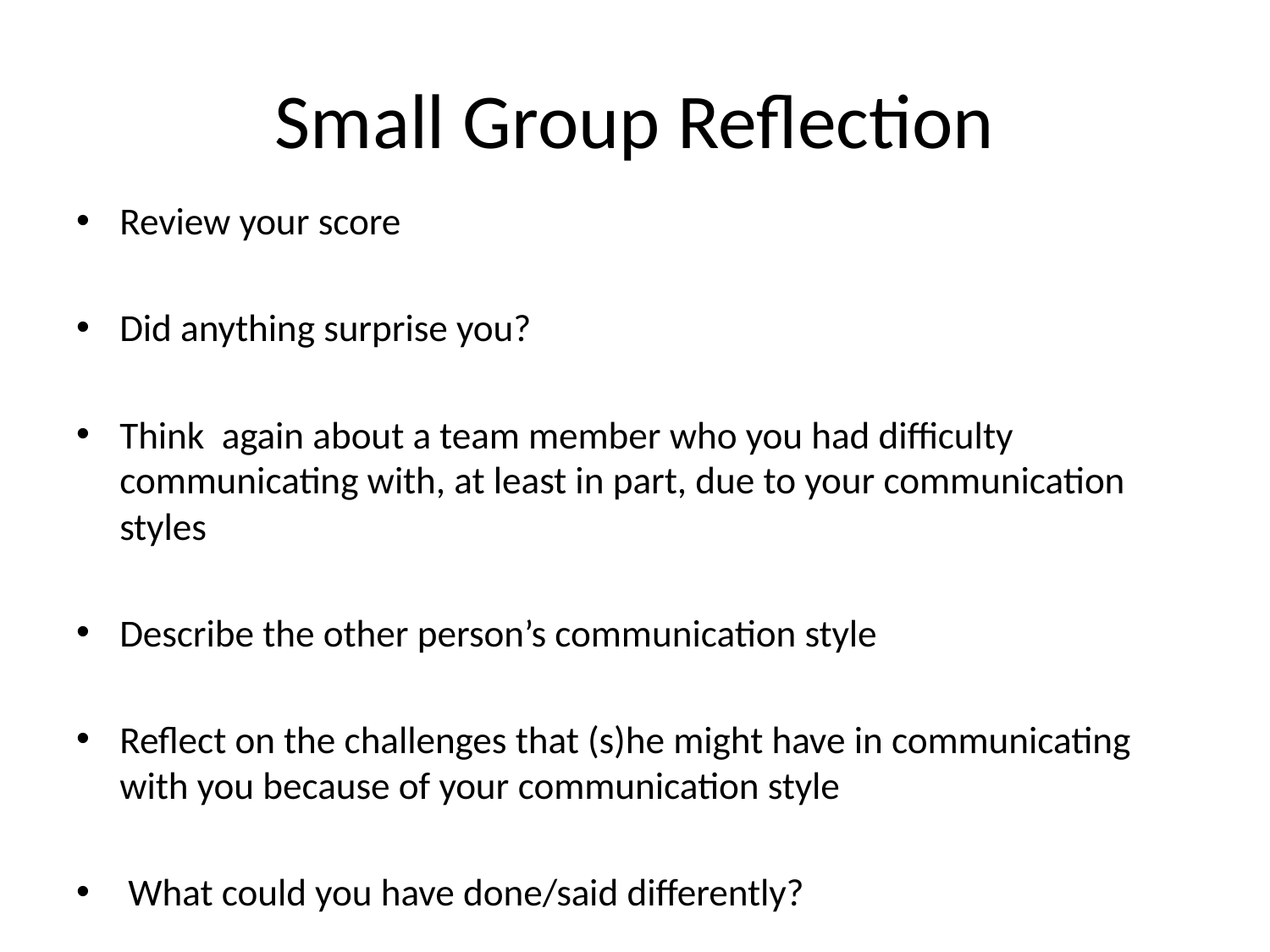

# Small Group Reflection
Review your score
Did anything surprise you?
Think again about a team member who you had difficulty communicating with, at least in part, due to your communication styles
Describe the other person’s communication style
Reflect on the challenges that (s)he might have in communicating with you because of your communication style
 What could you have done/said differently?

## Slide 26
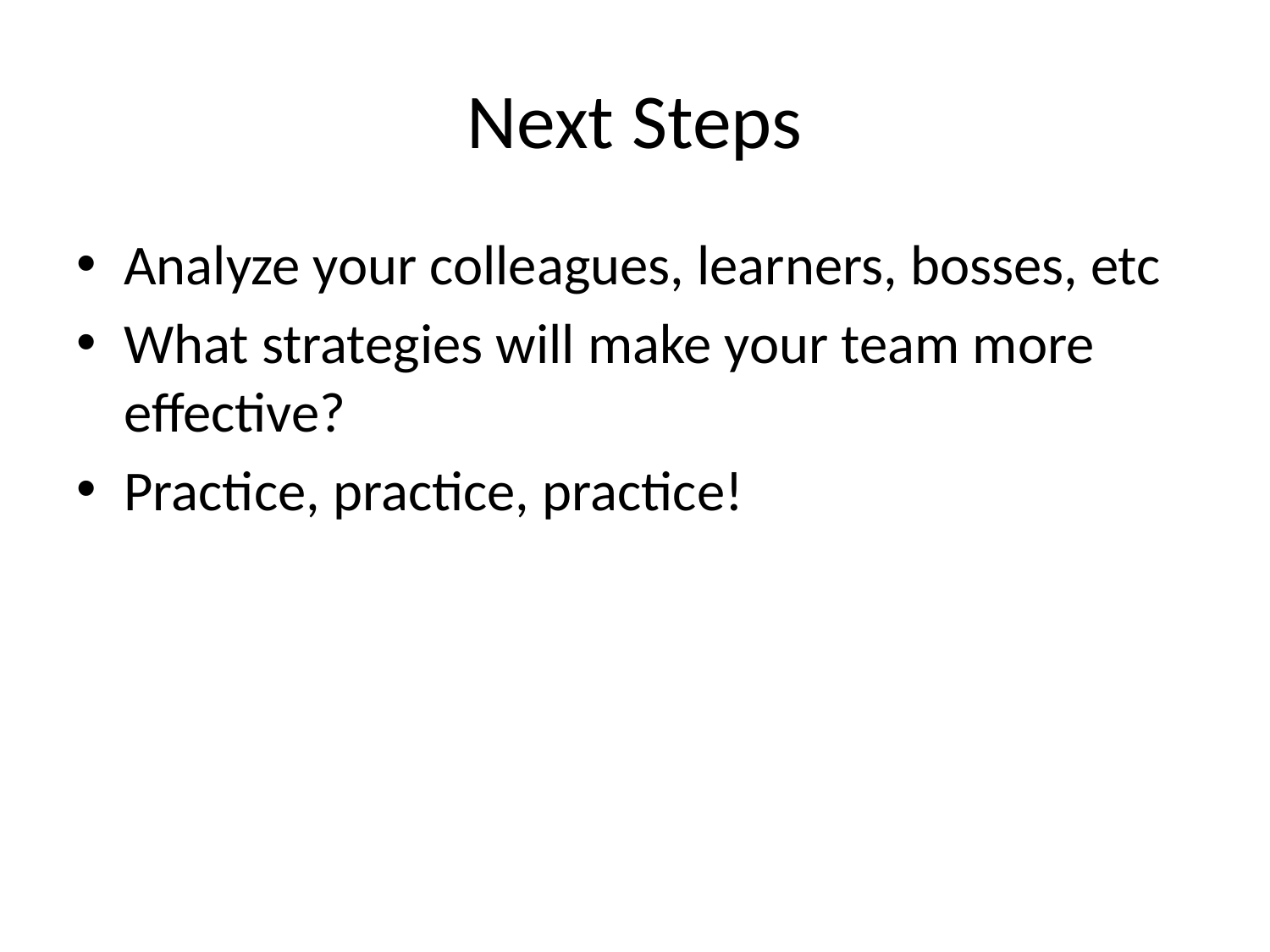

# Next Steps
Analyze your colleagues, learners, bosses, etc
What strategies will make your team more effective?
Practice, practice, practice!

## Slide 27
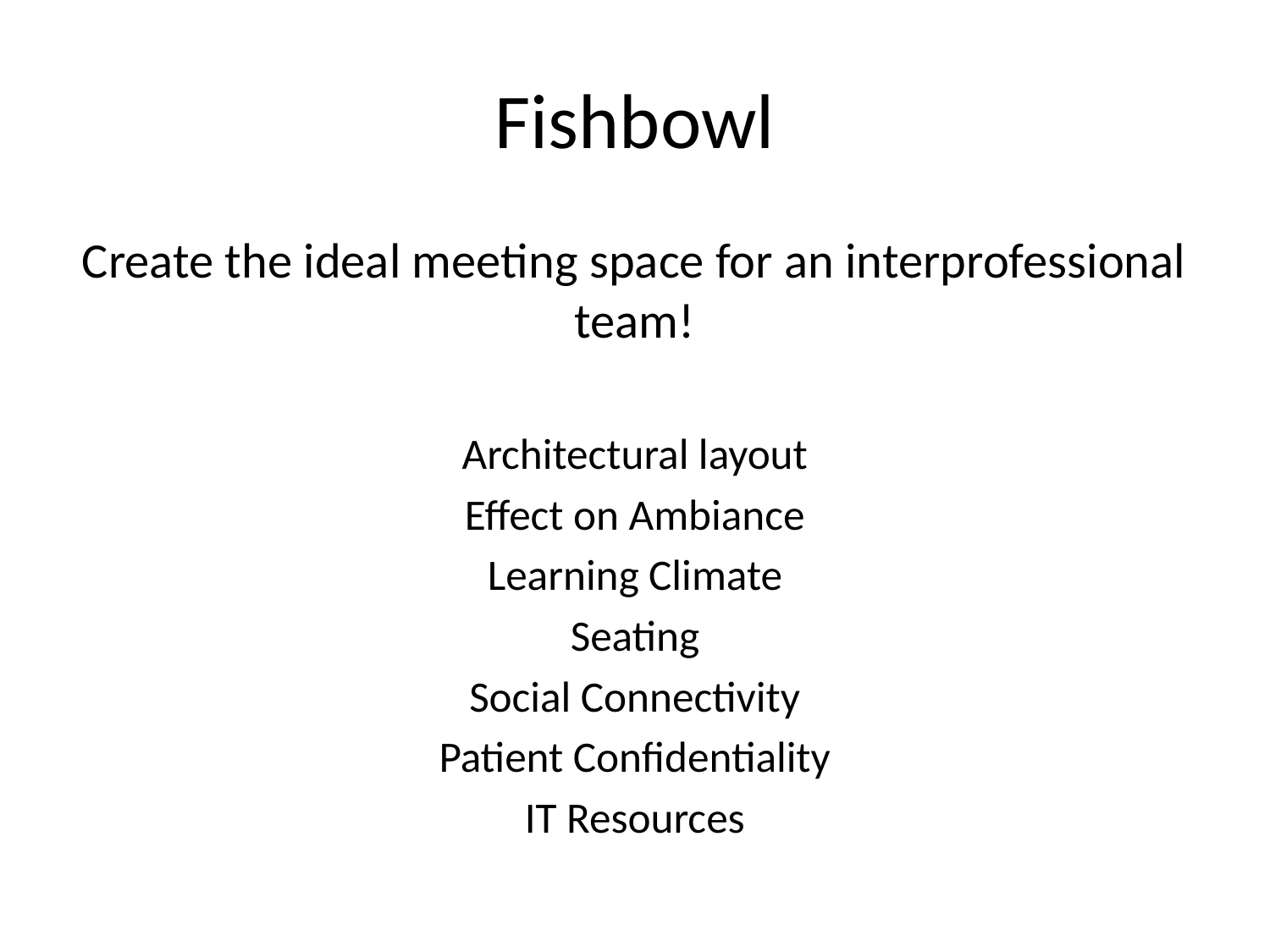

# Fishbowl
Create the ideal meeting space for an interprofessional team!
Architectural layout
Effect on Ambiance
Learning Climate
Seating
Social Connectivity
Patient Confidentiality
IT Resources

## Slide 28
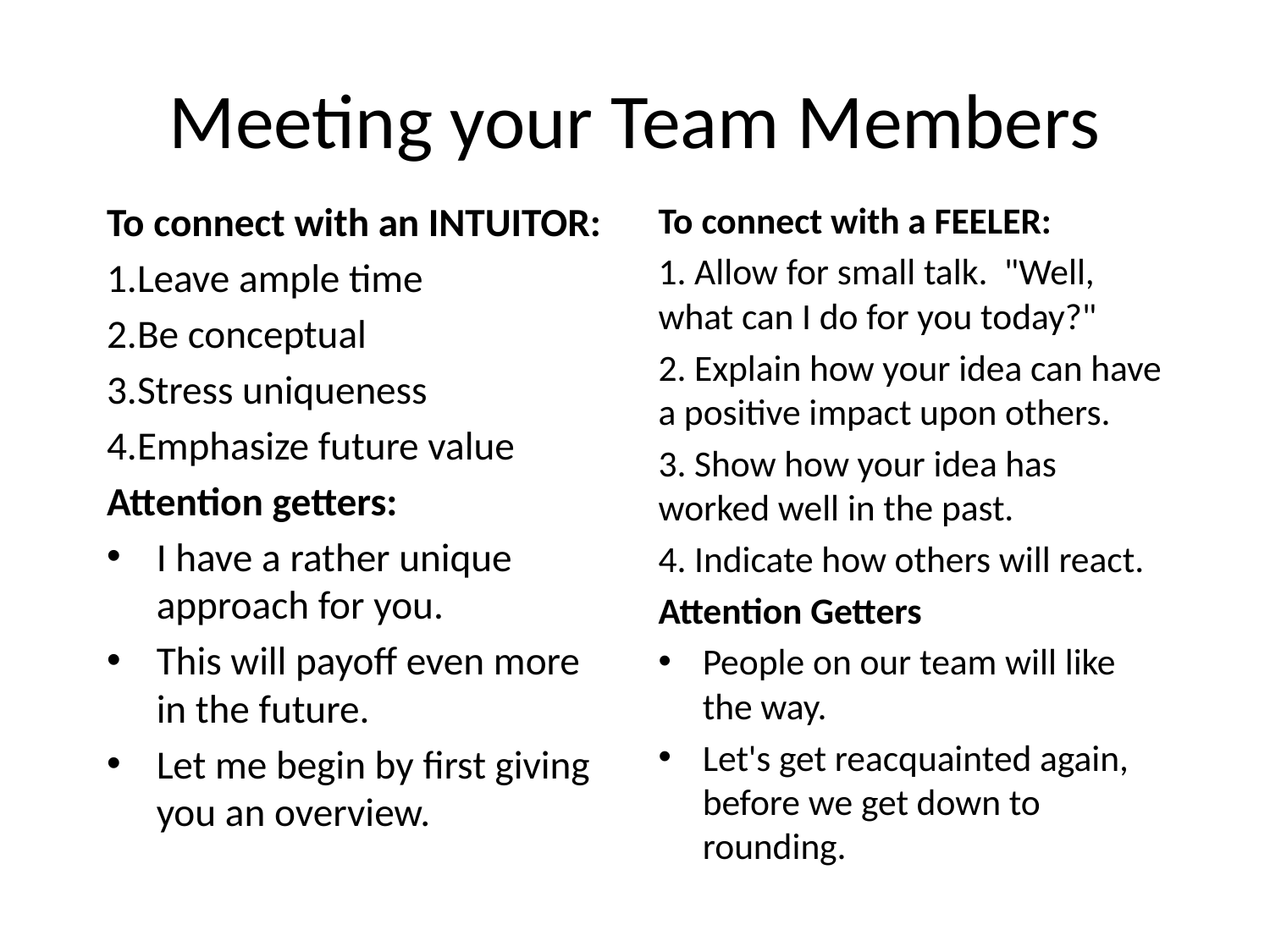

# Meeting your Team Members
To connect with an INTUITOR:
1.Leave ample time
2.Be conceptual
3.Stress uniqueness
4.Emphasize future value
Attention getters:
I have a rather unique approach for you.
This will payoff even more in the future.
Let me begin by first giving you an overview.
To connect with a FEELER:
1. Allow for small talk. "Well, what can I do for you today?"
2. Explain how your idea can have a positive impact upon others.
3. Show how your idea has worked well in the past.
4. Indicate how others will react.
Attention Getters
People on our team will like the way.
Let's get reacquainted again, before we get down to rounding.

## Slide 29
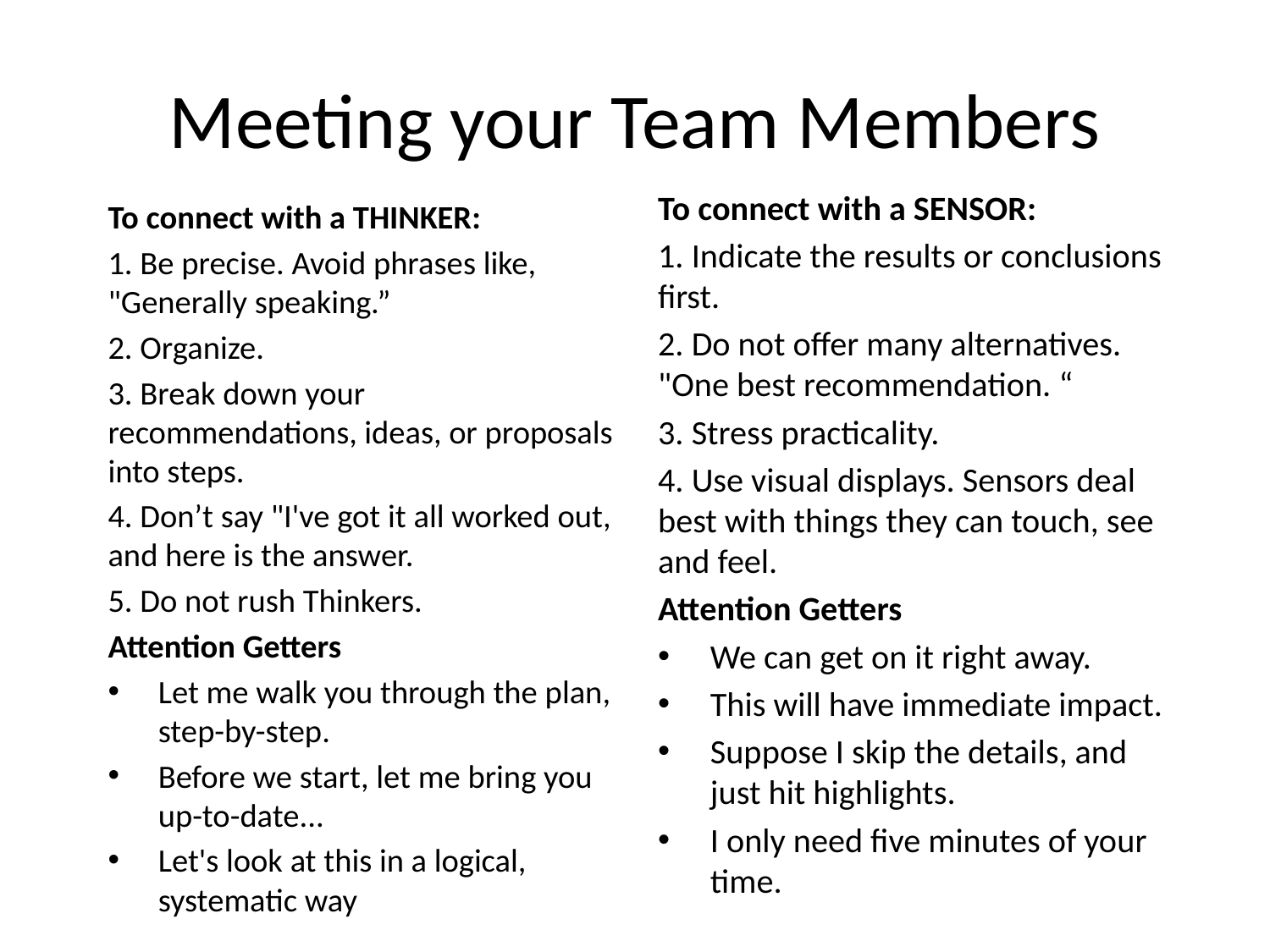

# Meeting your Team Members
To connect with a SENSOR:
1. Indicate the results or conclusions first.
2. Do not offer many alternatives. "One best recommendation. “
3. Stress practicality.
4. Use visual displays. Sensors deal best with things they can touch, see and feel.
Attention Getters
We can get on it right away.
This will have immediate impact.
Suppose I skip the details, and just hit highlights.
I only need five minutes of your time.
To connect with a THINKER:
1. Be precise. Avoid phrases like, "Generally speaking.”
2. Organize.
3. Break down your recommendations, ideas, or proposals into steps.
4. Don’t say "I've got it all worked out, and here is the answer.
5. Do not rush Thinkers.
Attention Getters
Let me walk you through the plan, step-by-step.
Before we start, let me bring you up-to-date...
Let's look at this in a logical, systematic way

## Slide 30
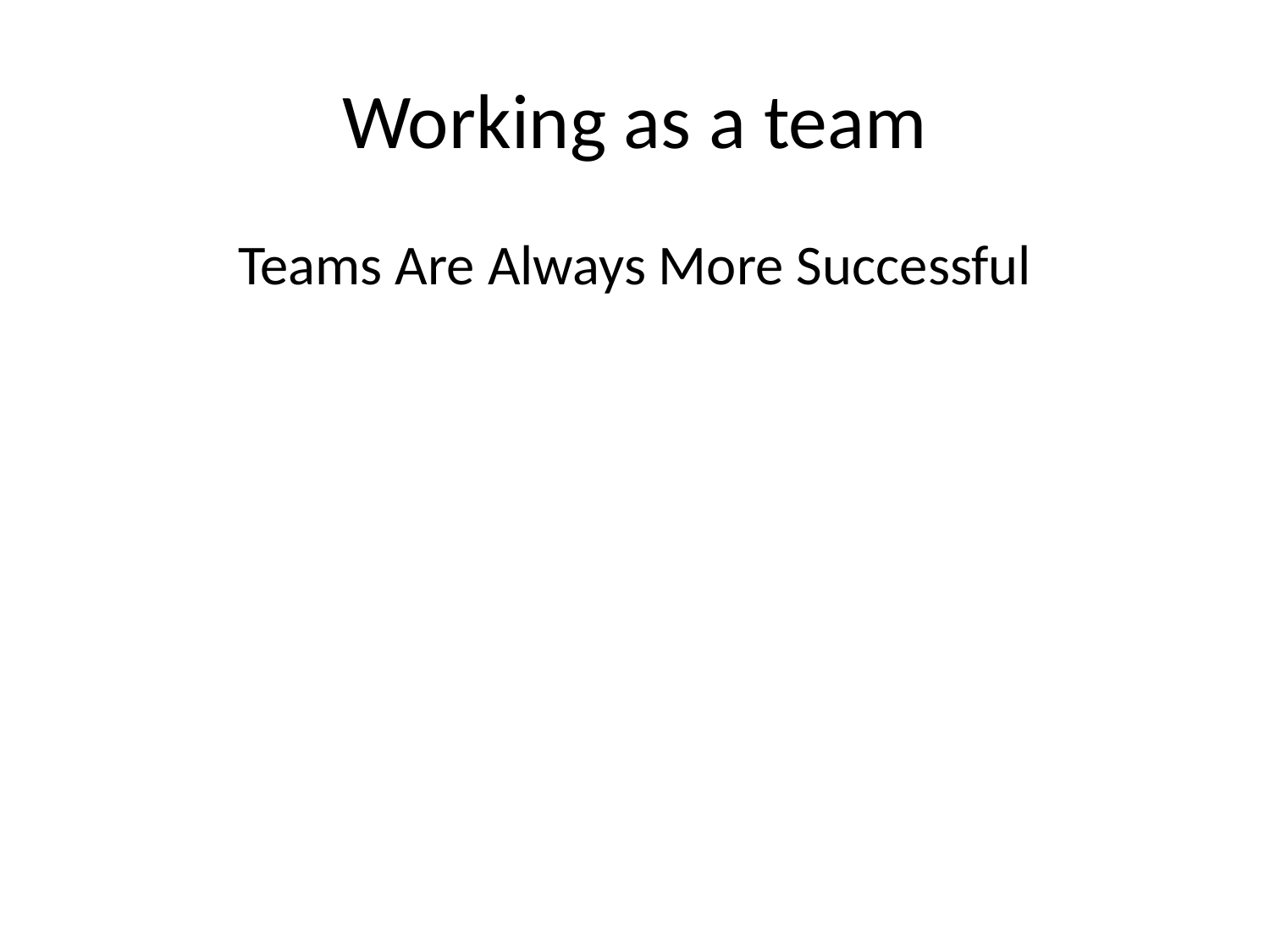

# Working as a team
Teams Are Always More Successful

## Slide 31
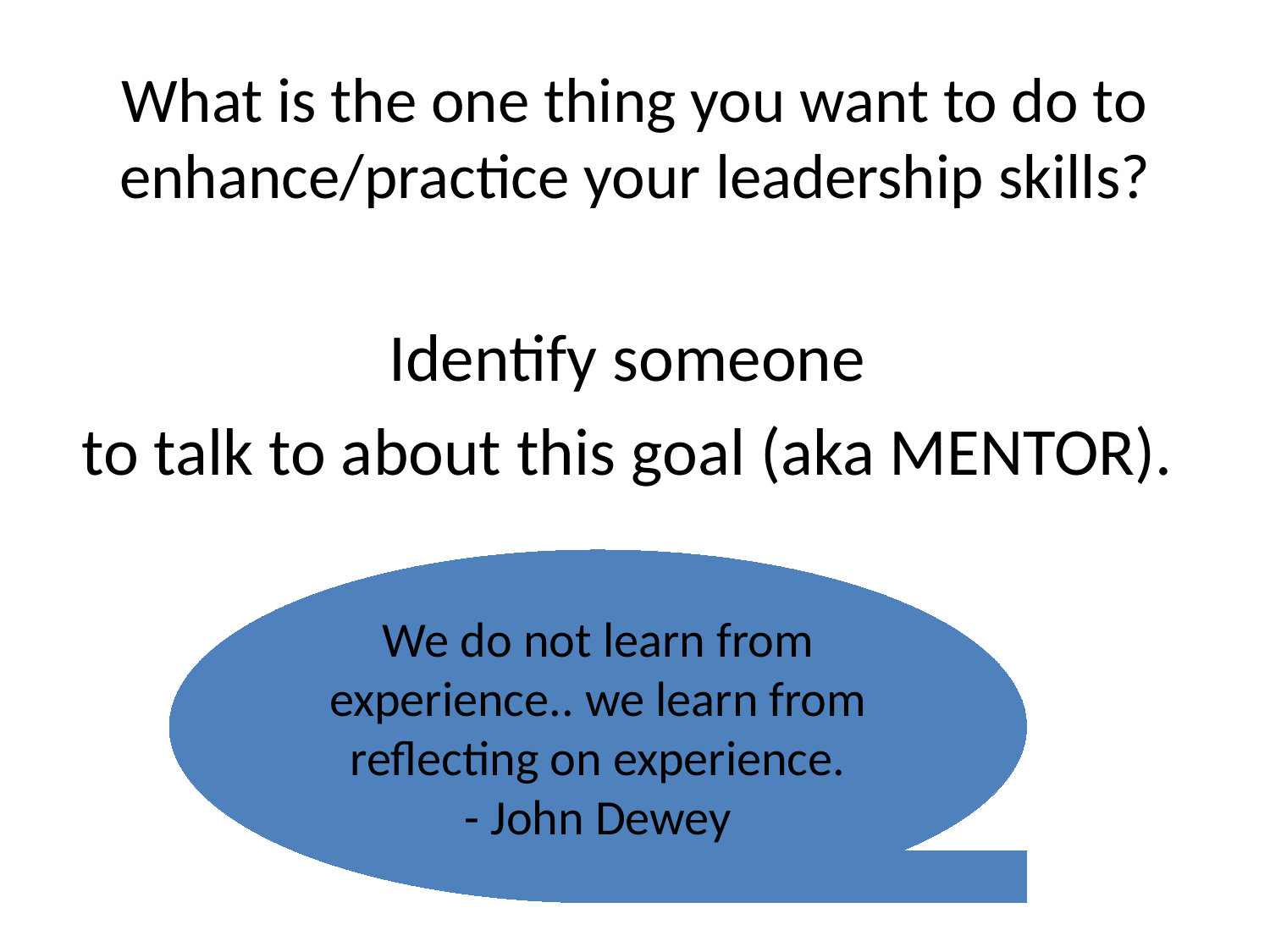

What is the one thing you want to do to enhance/practice your leadership skills?
Identify someone
to talk to about this goal (aka MENTOR).
We do not learn from experience.. we learn from reflecting on experience.
- John Dewey

## Slide 32
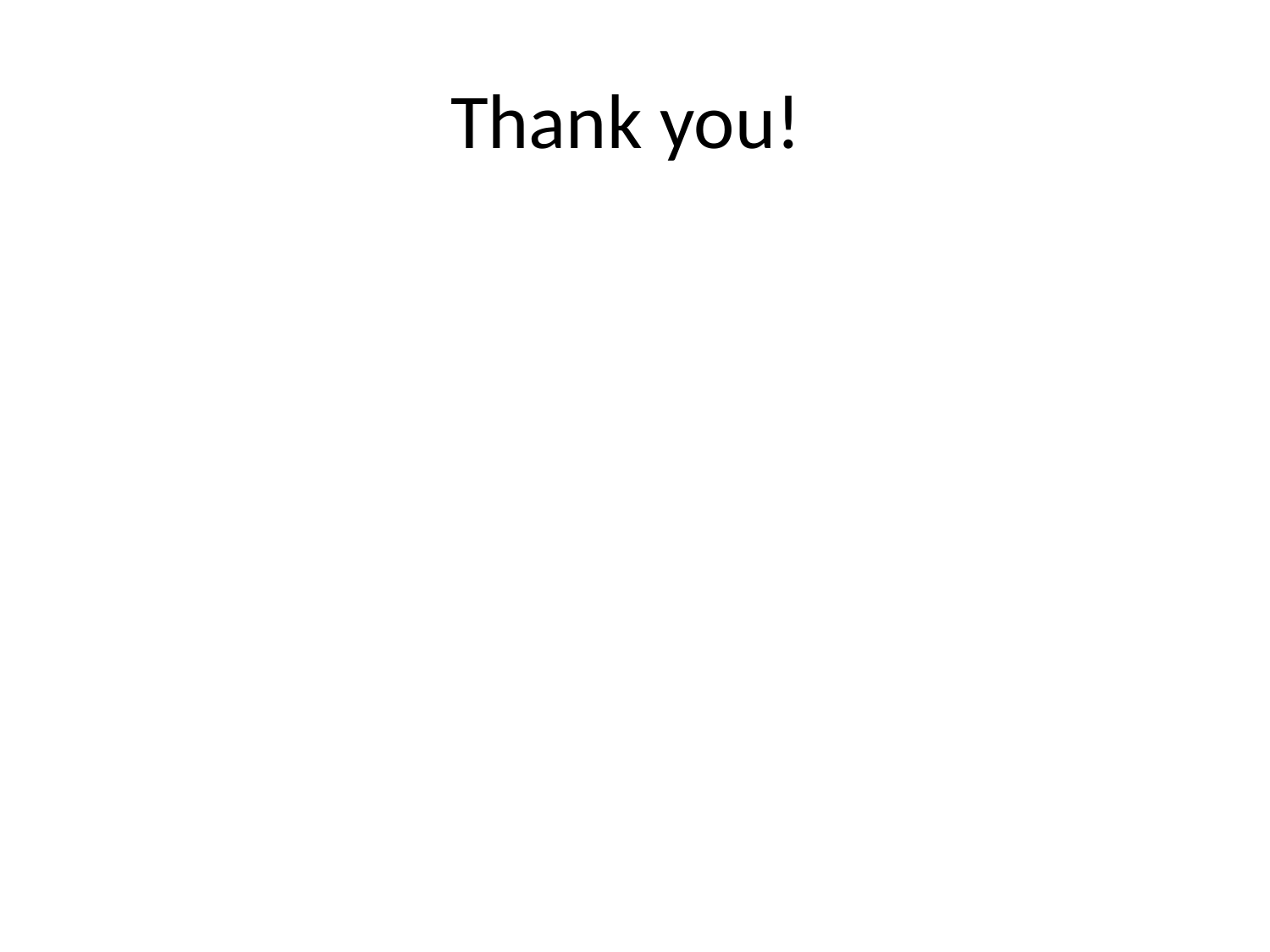

# Thank you!
