# Supplementary material for: Interprofessional Leadership Development: Role of Emotional Intelligence and Communication Skills Training
Source: MedEdPORTAL. 2022 May 13;18:11247. doi: 10.15766/mep_2374-8265.11247 (PMC9098732; doi:10.15766/mep_2374-8265.11247)
Supplement: Supplementary file 1 — EI in Interprofessional Leadership.pptxFacilitator Guide to Fishbowl Activity.docxSmall-Group Fishbowl Activity Evaluation.docxWorkshop Evaluation.docx [file mep_2374-8265.11247-s001.zip › C. Small-Group Fishbowl Activity Evaluation.docx]

**Appendix C: Small Group Fishbowl Activity: Observer Checklist**

**Team Dynamics/Communication**

|  | **Yes** | **No** | **By Which Profession** |
| --- | --- | --- | --- |
| A leader evolved from the group; if yes discipline? |  |  |  |
| Purpose of the meeting is clarified?  If not, what discipline(s) did not participate? |  |  |  |
| It appeared that team members felt valued for their contributions |  |  |  |
| Listening by team members was evident |  |  |  |
| Non-leaders felt secure in presenting thoughts/ideas |  |  |  |
| Team Conversation is clear/unambiguous-focused on goal of the meeting |  |  |  |
| Conflicting ideas are managed by the leader and/or team members |  |  |  |
| Individual role of team members are recognized |  |  |  |
| Closure of meeting occurred  -Directed by who? |  |  |  |
| Goals and accomplishments are re-iterated at closure  -Directed by who? |  |  |  |

**Identify if these Emotional Intelligence Skills were demonstrated in the team discussion:**

|  | **Yes** | **No** | **By Which Profession** |
| --- | --- | --- | --- |
| **Self-Awareness:** leader/team demonstrated self-confidence (self and team members) |  |  |  |
| **Self-regulation:** leader/team controlled disruptive emotions/impulses |  |  |  |
| **Motivation:** leader/team guided team members to achieve goal |  |  |  |
| **Empathy:** leader/team considered feelings of other team members |  |  |  |
| **Social Skills:** leader/team was able to move participants in right direction |  |  |  |
